# Transcriptomic changes in the plant pathogenic fungus *Rhizoctonia solani* AG-3 in response to the antagonistic bacteria *Serratia proteamaculans* and *Serratia plymuthica*

**DOI:** 10.1186/s12864-015-1758-z

**Published:** 2015-08-22

**Authors:** Konstantia Gkarmiri, Roger D. Finlay, Sadhna Alström, Elizabeth Thomas, Marc A. Cubeta, Nils Högberg

**Affiliations:** Department of Forest Mycology and Plant Pathology, Uppsala BioCenter, Swedish University of Agricultural Sciences, Box 7026, SE-75007 Uppsala, Sweden; Department of Plant Pathology, Center for Integrated Fungal Research, Fungal Disease Ecology, Genetics and Population Biology, North Carolina State University, 851 Main Campus Drive, Suite 233, 225 Partners III, Raleigh, NC 27606 USA

**Keywords:** *Rhizoctonia solani* (AG3 Rhs1AP), *Serratia proteamaculans* (S4), *Serratia plymuthica* (AS13), Biological control, Bacterial-fungal interactions, RNA sequencing, Transcriptome, Fungal gene expression

## Abstract

**Background:**

Improved understanding of bacterial-fungal interactions in the rhizosphere should assist in the successful application of bacteria as biological control agents against fungal pathogens of plants, providing alternatives to chemicals in sustainable agriculture. *Rhizoctonia solani* is an important soil-associated fungal pathogen and its chemical treatment is not feasible or economic. The genomes of the plant-associated bacteria *Serratia proteamaculans* S4 and *Serratia plymuthica* AS13 have been sequenced, revealing genetic traits that may explain their diverse plant growth promoting activities and antagonistic interactions with *R. solani*. To understand the functional response of this pathogen to different bacteria and to elucidate whether the molecular mechanisms that the fungus exploits involve general stress or more specific responses, we performed a global transcriptome profiling of *R. solani* Rhs1AP anastomosis group 3 (AG-3) during interaction with the S4 and AS13 species of *Serratia* using RNA-seq.

**Results:**

Approximately 104,504 million clean 75-100 bp paired-end reads were obtained from three libraries, each in triplicate (AG3-Control, AG3-S4 and AG3-AS13). Transcriptome analysis revealed that approximately 10 % of the fungal transcriptome was differentially expressed during challenge with *Serratia*. The numbers of S4- and AS13-specific differentially expressed genes (DEG) were 866 and 292 respectively, while there were 1035 common DEGs in the two treatment groups. Four hundred and sixty and 242 genes respectively had values of log_2_ fold-change > 3 and for further analyses this cut-off value was used. Functional classification of DEGs based on Gene Ontology enrichment analysis and on KEGG pathway annotations revealed a general shift in fungal gene expression in which genes related to xenobiotic degradation, toxin and antioxidant production, energy, carbohydrate and lipid metabolism and hyphal rearrangements were subjected to transcriptional regulation.

**Conclusions:**

This RNA-seq profiling generated a novel dataset describing the functional response of the phytopathogen *R. solani* AG3 to the plant-associated *Serratia* bacteria S4 and AS13. Most genes were regulated in the same way in the presence of both bacterial isolates, but there were also some strain-specific responses. The findings in this study will be beneficial for further research on biological control and in depth exploration of bacterial-fungal interactions in the rhizosphere.

**Electronic supplementary material:**

The online version of this article (doi:10.1186/s12864-015-1758-z) contains supplementary material, which is available to authorized users.

## Background

Soil microorganisms are key determinants of soil fertility and plant health. Terrestrial bacteria and fungi have undergone evolution and niche differentiation and interact in different ways with various outcomes in soil ecosystems. The presence of fungi may have diminished available ecological niches for bacteria, but can also result in the creation of new niches [9]. Greater insight into the mechanisms underlying natural antagonistic interactions between bacteria and fungi and the particular organisms involved has the potential to yield significant knowledge that can be used in the application of microorganisms for the biological control of plant diseases, complementing or replacing traditional chemical treatments.

Fungal responses to bacteria have been mainly studied within the context of interactions between ectomycorrhizal fungi with “mycorrhizal helper bacteria” [24]. Interactions of the ectomycorrhizal fungus *Amanita muscaria* with *Streptomyces* AcH 505 have been reported to promote fungal growth with changes in fungal metabolism, signalling, cell structure and cell growth response [60]. Regulation of genes involved in recognition processes, transcription and primary metabolism has been observed during confrontation of *Laccaria bicolor* with *Pseudomonas fluorescens*. Some of the molecular determinants were specific to the model organism used in the study, whereas others were also regulated in the same way by other rhizobacteria [17].

Most previous biological control studies have focused on the behaviour and mechanisms that beneficial biocontrol organisms utilize against fungal pathogens. Bacterial species belonging to the genera *Bacillus*, *Burkholderia*, *Collimonas* and *Pseudomonas* have been studied extensively from a biocontrol perspective [6, 30, 39, 70] and found to use a wide array of mechanisms influencing plant growth and health (reviewed by [7, 71]).

However responses of fungal pathogens to antagonistic bacteria have received much less attention. The simultaneous transcriptional profiling of the mycophagous bacterium *Collimonas fungivorans* and the fungus *Aspergillus niger* during interaction in *in-vitro* dual-culture assays has been reported [42]. Complex interactions related to antibiosis, trophism and nutrient competition were observed in both partners. Hyphal deformations of *Aspergillus* due to the presence of the bacteria were observed, coinciding with altered expression of genes related to lipid and cell wall degradation, cell defence and nitrogen deficiency.

Plant pathogens are also capable of exploiting a wide array of mechanisms in order to counteract and compete against antagonism from both microbial antagonists and other pathogens [19]. A more comprehensive mechanistic understanding of the molecular communication between pathogens and biological control agents in the rhizosphere is therefore needed to elucidate these complex interactions. There may be many different genetically regulated responses during the antagonist-pathogen interaction under the influence of different agents of stress on both sides (pathogen or antagonist), especially in the zone of interaction. Processes related to lipid-, carbon-, cell membrane- and cell wall metabolism, cell defence, self protection against oxidative stress, transcription and pathogenesis are transcriptionally regulated in fungi interacting with antagonistic microorganisms [33, 58, 59]. Additionally, fungi engaged in competition with antagonistic microorganisms frequently produce secondary metabolites, extracellular phenol-oxidizing enzymes, and differentiated structures in the zone of conflict [20, 28],

One important pathogen of several agricultural crops, including oilseed rape and potato, is the soil-borne basidiomycete fungus *Rhizoctonia solani*, (teleomorph *Thanatephorus cucumeris*) that causes damping off and root rot in oilseed rape and black scurf diseases of potato. Once established it is difficult to control because of its broad host range, saprophytic life style and persistence of the sclerotia and mycelium in soil and plant material. Potato is mostly affected by AG3 isolates [2], especially in the potato production areas under cool climates such as northern Europe [37]. Existing control strategies based on chemical treatment and crop cultivation practices have limited efficacy and alternative biocontrol strategies are required to improve sustainability [56].

Plant-associated bacteria including those belonging to *Serratia,* have been screened for antagonism against plant pathogenic fungi and have been shown to be beneficial in oilseed rape [1]. The genomes of four *Serratia* strains (AS9, AS12, AS13 and S4) have been recently sequenced [49–52]. The genomes of S4 and AS13 have been studied in detail and differences in their type secretion systems, colonization patterns, antibiotic and secondary metabolites production have been demonstrated that are consisten with their diverse plant growth promoting activities and antagonistic interactions with the plant pathogenic fungus *R. solani* AG2-1 [47]. *In-vitro* experiments with bacterial strains AS13 and S4 challenged with *R. solani* AG2-1 revealed greater antagonistic activity of S4 compared to the activity of AS13. Transcriptomic analyses of these two bacterial strains inoculated with the pathogen in *in-vitro* systems revealed different patterns of gene expression compared to the non-inoculated control treatments [47, 48].

The aim of the present study was to elucidate the molecular mechanisms by which the fungus *R. solani* responds to challenge with the plant-associated *Serratia* bacteria S4 and AS13, exhibiting different levels of antagonism. We hypothesized that there would be a greater rearrangement of the fungal transcriptome in response to the stronger antagonist S4 than to AS13. We also expected that some general changes in metabolism would be involved in fungal stress responses to both bacteria, but that specific responses to individual bacterial strains would also occur. To test this hypothesis an *in-vitro* confrontation assay was developed and RNA sequencing of the pathogenic fungus was performed by exploiting the recently published *R. solani* Rhs1AP AG3 genome [16], accession number [GenBank: JATN00000000]. At the gene transcript level, the overall fungal response to the bacteria was similar for both S4 and AS13 and large-scale changes in the fungal transcriptome were evident suggesting the simultaneous alteration of primary metabolism, activation of defence and attack mechanisms and shifts in hyphal morphology and growth.

## Results

### Effects of *Serratia* on growth and morphology of *R. solani* mycelium

**Fig. 1:**
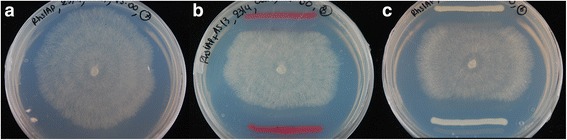
Dual culture *in vitro* bacterial-fungal assays. **a** Control *R. solani* monoculture. **b** *R. solani* challenged with *Serratia proteamaculans* S4. **c** *R. solani* challenged with *Serratia plymuthica* AS13

**Fig. 2:**
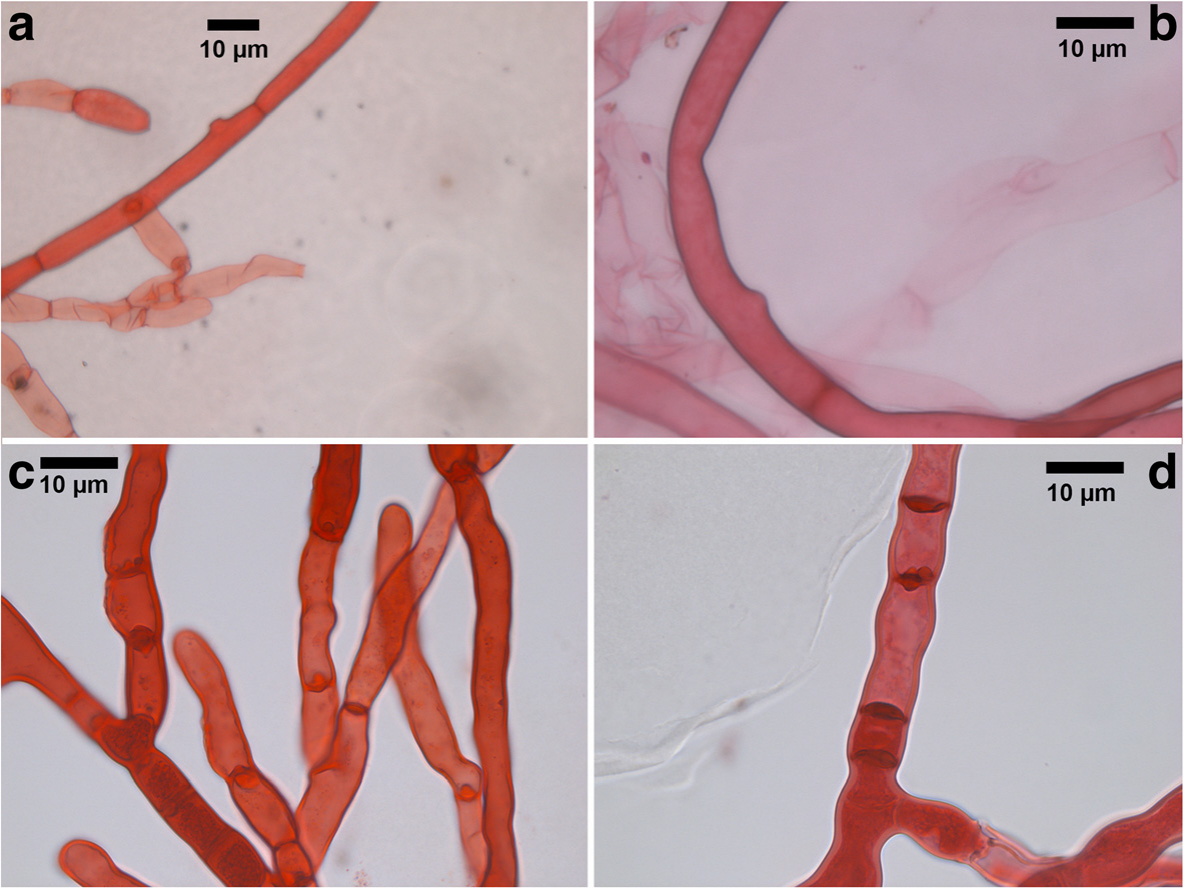
**a**, **b** *R. solani*: straight mycelium, normal branching, normal septation. **c**, **d** *R. solani* challenged with *Serratia plymuthica.* Note increased frequency of septa and branching, swollen mycelium and dolipore septa, cell wall thickening

When *R. solani* was challenged with *Serratia* S4 and AS13, clear inhibition of fungal mycelial growth was evident already at 72 hours in both cases compared to the control treatments without bacteria (Fig. 1). This inhibition persisted for at least four weeks (data not shown). Microscopic observations of fungal hyphae during interactions with *Serratia* revealed swollen mycelium, with increased septation and branching and thickened cell walls compared to control straight mycelium with normal branching and septation (Fig. 2).

### Bioinformatic analysis

#### Read trimming and mapping of Illumina reads

To examine the molecular response of *R. solani* to *S. proteamaculans* S4 and *S. plymuthica* AS13, we compared the transcriptome of the fungus at 72 h post inoculation when grown in monoculture with those when grown during interaction with each of the two bacteria. Nine libraries were created in total for deep sequencing *R. solani* AG3-Control, *R. solani* AG3-S4, and *R. solani* AG3-AS13, each in triplicate) and the raw reads (100 bp, single-end) obtained from Illumina® TruSeq were firstly filtered. The number of contaminant-free filtered reads ranged from 9 to 12 million reads per sample (Table 1). They were mapped to the Rhs1AP reference genome using Bowtie2 and Tophat2 [GenBank: JATN00000000]. After quality trimming 104,504,819 sequence reads of average length 75-100 bp were obtained and used to align to the reference genome. Alignment details are shown in Table 1. Box plot analysis of fragments per kilobase of exon per million fragments mapped (FPKM) distributions of the nine samples in our study revealed that the transcripts had similar distribution, suggesting that the sets of sequence data are comparable and suitable for our downstream analyses (Additional file 1: Figure S1). Additionally, Principal Component Analysis (PCA) of the log_2_-transformed FPKM values highlighted the difference between the samples and revealed that presence of both bacterial strains S4 and AS13 affected the fungal transcriptome (Additional file 2: Figure S2).

**Table 1:** Summary of read numbers and alignment based on the RNA-Seq data. C = control, S4 = challenged with *Serratia proteamaculans*, AS13 = challenged with *Serratia plymuthica,* R1, R2, R3 are three biological replicates

Sample	Total input reads	Left mapped reads	Left mapped reads with multiple alignments	Right mapped reads	Right mapped reads with multiple alignments	Total aligned pairs	Concordant pair alignment rate
C_R1	11,095,432	9,331,561 (84.1 %)	874,389 (9.4 %)	9,329,491 (84.1 %)	876,749 (9.4 %)	9,094,851	81.9 %
C_R2	11,494,727	9,617,041 (83.7 %)	860,263 (8.9 %)	9,616,430 (83.7 %)	862,125 (9.0 %)	9,363,011	81.4 %
C_R3	12,225,022	10,176,425 (83.2 %)	949,473 (9.3 %)	10,175,088 (83.2 %)	952,599 (9.4 %)	9,904,261	80.9 %
S4_R1	9,357,641	7,717,332 (82.5 %)	746,407 (9.7 %)	7,715,127 (82.4 %)	749,440 (9.7 %)	7,505,336	80.1 %
S4_R2	11,669,755	9,654,736 (82.7 %)	983,020 (10.2 %)	9,653,409 (82.7 %)	985,993 (10.2 %)	9,394,327	80.4 %
S4_R3	11,739,231	9,611,162 (81.9 %)	934,484 (9.7 %)	9,609,086 (81.9 %)	936,772 (9.7 %)	9,355,791	79.6 %
AS13_R1	12,188,851	10,163,886 (83.4 %)	1,032,695 (10.2 %)	10,160,009 (83.4 %)	1,034,346 (10.2 %)	9,893,969	81.1 %
AS13_R2	12,456,782	10,346,804 (83.1 %)	968,441 (9.4 %)	10,346,410 (83.1 %)	969,736 (9.4 %)	10,078,070	80.8 %
AS13_R3	12,277,378	10,116,891 (82.4 %)	945,375 (9.3 %)	10,115,612 (82.4 %)	951,473 (9.4 %)	9,856,584	80.2 %

#### Effects of *Serratia* S4 and AS13 on gene expression of *R. solani*

Approximately 10 % of the whole fungal transcriptome was differentially expressed with a total of 1901 and 1327 genes significantly up- or down- regulated for S4 and AS13 respectively (Additional file 3: Figure S3). The numbers of S4- and AS13-specific differentially expressed genes (DEGs) were 866 and 292 respectively, while there were 1035 common DEGs in the two treatment groups Additional file 4: Table S1, Additional file 5: Table S2. A total of 460 and 242 genes respectively had fold values exceeding +/− 8x and this cut-off value was used for all further analyses, unless otherwise specified.

Following differential expression analysis, the transcripts with fold values exceeding log_2_(3) (in total 702 sequences) were subjected to BLASTx. Of those, 674 sequences were finally successfully annotated after enhancing the annotation by including the results of an InterproScan database search and the ANNEX augmentation procedure [26].

The top 20 differentially expressed genes of *R. solani* during interaction with S4 and AS13 are presented in Tables 2 and 3. Highly upregulated genes included aliphatic nitrilase, betaine lipid, isocitrate lyase, hydrolases, ricin-like lectins, proteins of cytochrome P450, glutamine amidotransferase, glutathione s-transferase, haloacid dehalogenase and transporters. Highly downregulated genes included a glycoside hydrolase family 61 protein, a copper-centre containing protein, a tyrosinase tyrosinase central domain protein, proteins of cytochrome P450 and pectinesterases and xylanase, genes related to plant cell wall modification and degradation.

**Table 2:** Top 20 upregulated genes of *Rhizoctonia solani* when challenged with *Serratia proteamaculans* S4 or *Serratia plymuthica* AS13

Gene Id	Putative function	log2 fold change
S4 Up		
1.t002520	aliphatic nitrilase	6.92835
17.t000054	betaine lipid	6.91571
1.t001569	l-psp endoribonuclease family protein	6.39109
1.t001237	dienelactone hydrolase family protein	5.94347
11.t000148	hypothetical protein RSOL_500330	5.92287
1.t001998	mfs general substrate transporter	5.89682
3.t000606	response regulator receiver domain protein	5.76394
4.t000541	hypothetical protein RSOL_421740	5.71167
4.t000088	kinase domain protein	5.45440
4.t000274	isocitrate lyase	5.28342
1.t000080	putative hydrolase	5.26161
1.t003583	epoxide hydrolase	5.26161
6.t000086	haloacid dehalogenase	5.23554
1.t004221	glutathione s-transferase c-terminal-like protein	4.84274
5.t000634	acyltransferase ctase cot cpt	4.82218
1.t003262	glutathione s-transferase	4.82154
1.t000120	tlc domain protein	4.33277
1.t001973	cytochrome p450 family protein	4.24327
3.t000070	secreted protein	4.08181
12.t000229	ricin b-like lectin	4.05721
AS13 Up		
17.t000054	betaine lipid	7.56872
3.t000070	secreted protein	4.98602
1.t003006	inorganic phosphate transporter	4.74944
4.t000445	ricin-type beta-trefoil lectin omain-containing protein	4.52512
1.t000120	tlc domain protein	4.17569
1.t001562	glycoside hydrolase family 13 protein	4.10199
3.t000380	nad h-binding family protein	3.94301
1.t002656	alpha amylase	3.93229
2.t000151	hypothetical protein RSOL_273340	3.90046
219.t000001	helix loop helix dna-binding domain partial	3.89048
1.t002794	guanyl-specific ribonuclease f1	3.87433
10.t000302	fad-binding domain protein	3.75220
1.t001394	plc-like phosphodiesterase	3.53473
1.t002520	aliphatic nitrilase	3.51621
9.t000274	hypothetical protein RSOL_481390	3.49204
4.t000274	isocitrate lyase	3.45076
5.t000433	nad -binding protein	3.44690
11.t000148	hypothetical protein RSOL_500330	3.38336
1.t004152	hypothetical protein RSOL_034560. partial	3.37974
1.t004112	gdsl-like lipase partial	3.22982

**Table 3:** Top 20 down-regulated genes of *Rhizoctonia solani* when challenged with *Serratia proteamaculans* S4 or *Serratia plymuthica* AS13

Gene Id	Putative function	log2 fold change
S4 Down		
3.t000246	di-copper centre-containing protein	−6.01495
15.t000002	endo- -beta-xylanase	−3.89385
1.t002315	glycoside hydrolase family 61 protein	−3.84167
1.t003360	duf1620-domain-containing protein	−3.62845
1.t003607	carbohydrate-binding module family 1 partial	−3.59128
2.t001565	tyrosinase tyrosinase: common central domain protein	−3.23190
2.t001687	pci-domain-containing protein	−3.23190
6.t000189	cytochrome p450	−3.10930
1.t003681	transmembrane partial	−3.07966
4.t000240	cytochrome p450 family protein	−3.02703
2.t000265	glycoside hydrolase family 5 protein	−2.98684
6.t000299	pectinesterase short = pe	−2.96204
6.t000302	pectinesterase short = pe	−2.96204
2.t000715	glycoside hydrolase family 45 protein	−2.91018
6.t000588	pectin lyase	−2.90836
2.t000524	copper radical oxidase	−2.83510
2.t001146	transmembrane protein. putative	−2.68567
3.t000919	cytochrome p450 monooxygenase pc-bph	−2.67932
3.t000100	isoamyl alcohol	−2.65052
1.t003090	glycoside hydrolase	−2.64223
AS13 Down		
3.t000246	di-copper centre-containing protein	−3.33761
15.t000002	endo- -beta-xylanase	−2.96027
1.t000495	f-box-like domain	−2.67743
1.t002315	glycoside hydrolase family 61 protein	−2.61483
1.t001349	di-copper centre-containing protein	−2.55427
5.t000733	hypothetical protein RSOL_432000. partial	−2.55164
2.t001565	tyrosinase tyrosinase: common central domain protein	−2.45946
2.t001687	pci-domain-containing protein	−2.45946
6.t000588	pectin lyase	−2.45511
3.t000100	isoamyl alcohol	−2.42676
6.t000189	cytochrome p450	−2.39924
3.t000448	alcohol oxidase	−2.29475
1.t003607	carbohydrate-binding module family 1 partial	−2.26637
6.t000134	gnat family	−2.25863
2.t000714	glycoside hydrolase family 45 protein	−2.21676
2.t001146	transmembrane protein. putative	−2.20193
2.t000715	glycoside hydrolase family 45 protein	−2.19200
4.t000240	cytochrome p450 family protein	−2.16637
2.t001139	hypothetical protein RSOL_321070	−2.15247
2.t001148	hypothetical protein RSOL_321060	−2.15247

#### Functional classification of DEGs in *R. solani* influenced by *Serratia*

Enrichment analysis of treatment differences from the reference genome revealed significant over- and under-representation of different Gene Ontology (GO) terms.

#### Interactions with S4

 For the treatment with S4, 18 up-regulated GO terms were over- and 15 were under- represented. The over-represented terms corresponding to the lowest p-values in increasing order included GO:0016491 (oxidoreductase activity), GO:0004298 (threonine-type endopeptidase activity), GO:0055114 (oxidation-reduction process), GO:0009405 (pathogenesis) and the under-represented GO:0003677 (DNA binding), GO:0005840 (ribosome), GO:0006996 (organelle organization), GO:0060255 (regulation of macromolecule metabolic process) (Additional file 6: Figure S4a and b). Downregulated genes belong to nine enriched over-represented terms representatively including GO:0003723 (RNA-binding), GO:0008026 (ATP-dependent helicase activity), GO:0006397 (mRNA processing), GO:0006334 (nucleosome assembly) and 4 under-represented terms GO:0019538 (protein metabolic process), GO:0004175 (endopeptidase activity). GO:0003735 (structural constituent of ribosome) and GO:0016746 (transferase activity, transferring acyl groups) (Additional file 7: Figure S5a and b).

#### Interactions with AS13

For the *R. solani* challenged with AS13, 12 upregulated GO terms were over- and 17 were under-represented. The over-represented terms corresponding to the lowest p-values in increasing order included GO:0004298 (threonine-type endopeptidase activity), GO:0055114 (oxidation-reduction process), GO:0019773 (proteasome core complex, alpha-subunit complex), GO:0051603 (proteolysis involved in cellular protein catabolic process) and the under-represented GO:0003677 (DNA binding), GO:0017111 (nucleoside-triphosphatase activity), GO:0006996 (organelle organization), GO:0044428 (nuclear part) (Additional file 8: Figure S6a and b). Down-regulated genes belonged to eight enriched over-represented terms including GO:0008026 (ATP-dependent helicase activity), GO:0003723 (RNA binding), GO:0005730 (nucleolus), GO:0006334 (nucleosome assembly) (Additional file 9: Figure S7) and one under-represented term GO:0005737 (cytoplasm).

When comparing the enriched categories between the two treatments with S4 and AS13, some up-regulated GO terms such as threonine-type endopeptidase activity, oxidation-reduction process, pyridine-containing compound biosynthetic process, pyruvate metabolism, proteolysis involved in cellular protein catabolic process and proteasome core complex were over-represented in both cases. Terms under-represented in both cases included DNA-dependent transcription, purine ribonucleotide metabolic process, nucleoside-triphosphatase activity, ribosome biogenesis, organelle membrane, DNA metabolic process, organelle organization, ribosome and DNA binding (Additional file 3: Figures S3 and Additional file 7: Figure S5). The down-regulated GO terms nucleosome, nucleolus, nucleosome assembly, mRNA processing, ATP-dependent helicase activity and RNA binding were commonly over-represented in both treatments. Nucleotide binding and eukaryotic translation initiation factor 3 were over-represented only in the presence of S4, whereas DNA-directed RNA polymerase activity and cellular components integral to membrane were only over-represented in the presence of AS13 (Additional file 7: Figures S5 and Additional file 9: Figure S7).

 Analyses of over represented cellular locations of genes expressed differentially in response to S4 and AS13 are shown in Additional file 10: Figures S8 and Additional file 11: Figure S9 respectively. In the presence of both S4 and AS13, overrepresentation of upregulated genes localized integrally to the membrane and in the extracellular region was observed. Downregulated genes in the presence of S4 were mainly localized in the nucleus, whereas in the presence of AS13 there were 3 genes integral to the membrane, 3 in the extracellular region and 1 in the nucleus.

#### Effects of *Serratia* S4 and AS13 on KEGG pathways in *R. solani*

**Fig. 3:**
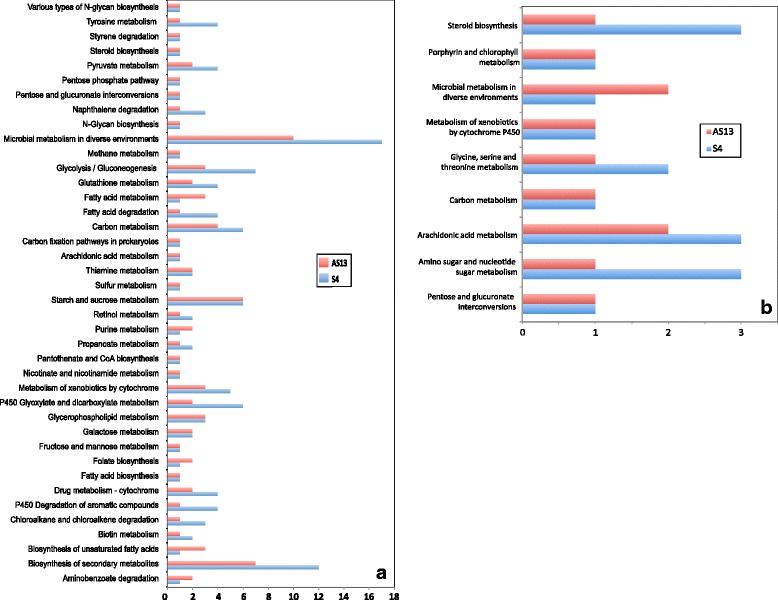
KEGG pathway annotations found to be common between the treatments with S4 *Serratia proteamaculans* and AS13 *Serratia plymuthica* for metabolism-related differentially expressed genes with fold values exceeding log_2_(3), (**a**) up-regulated genes, (**b**) down-regulated genes

**Fig. 4:**
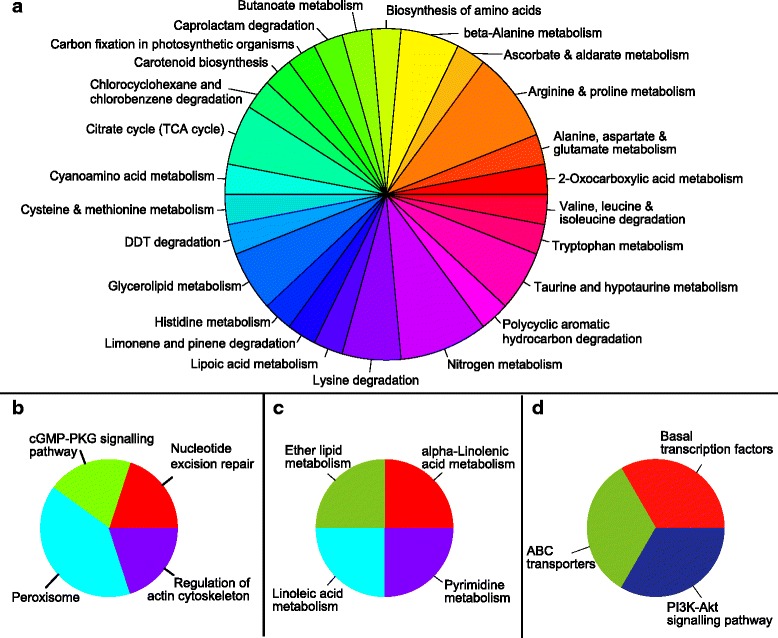
KEGG pathway annotations found to be unique for the treatments with **a**) S4 *Serratia proteamaculans* for metabolism-related, **b**) S4 *Serratia proteamaculans* for signalling-related, **c**) AS13 *Serratia plymuthica* for metabolism-related, **d**) AS13 *Serratia plymuthica* for signalling-related differentially expressed genes being up-regulated with fold values exceeding log_2_(3)

**Fig. 5:**
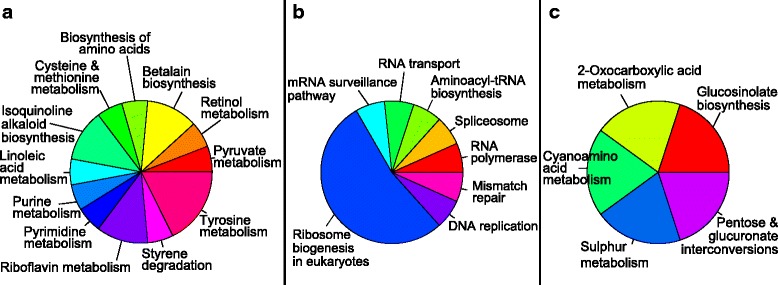
KEGG pathway annotations found to be unique for the treatments with **a**) S4 *Serratia proteamaculans* for metabolism-related, S4 *Serratia proteamaculans* for signalling-related, **b**) AS13 *Serratia plymuthica* for metabolism-related, **c**) AS13 *Serratia plymuthica* for signalling-related differentially expressed genes being down-regulated with fold values exceeding log_2_(3)

KEGG pathway annotations of DEGs ≥ log_2_(3) were obtained using the KAAS annotation server and revealed the presence of enzymes involved in taurine biosynthesis, glycerophospholipid metabolism, drug metabolism by cytochrome P450, sucrose metabolism, nicotinate metabolism, ascorbate metabolism, biosynthesis of unsaturated fatty acids, pyruvate and vitamin B6 metabolism. Even though the overall response of the fungus to both bacterial strains is similar, some KEGG pathways were present in only one of the treatments. Figure 3a and b display the number of metabolism-related genes for specific KEGG pathways that were found to be up- and down- regulated respectively in both treatments. When challenging *R. solani* with S4, there was greater restructuring of the fungal transcriptome than with AS13. Genes related to pyruvate, propanoate, methane, gluoxylate and glycerophospholipid metabolism, metabolism of xenobiotics by cytochrome P450, glucolysis, fatty acid and charoalcane degradation were prominent in the S4 treatment. Additionally there were 25 unique KEGG pathways obtained from annotations of genes being up-regulated only in challenge with S4 (Fig. 4a) and 4 unique ones found when AS13 was present (Fig. 4b). Twelve and 7 KEGG metabolic pathways were found to be uniquely downregulated in presence of S4 and AS13 respectively (Fig. 5a and b).

Apart from metabolism-related annotations, the KAAS server also provides annotations related to genetic and environmental information processing and cellular processes. Genes encoding for enzymes of the lysosomes, two component system and mitogen-activated protein kinases (MAPK) were found to be upregulated in the presence of both antagonistic bacteria. Genes involved in translation, transcription and repair and replication were commonly downregulated. Genes encoding for enzymes of peroxisomes, cGMP-PKG signalling, nucleotide excision repair and actin cytoskeleton regulation were found to be uniquely upregulated in the presence of S4. On the contrary, genes related to ribosome biogenesis in eukaryotes, mRNA surveillance, RNA transport, DNA replication and mismatch repair were downregulated only during confrontation with S4.

### Quantitative Real-Time PCR validation

**Fig. 6:**
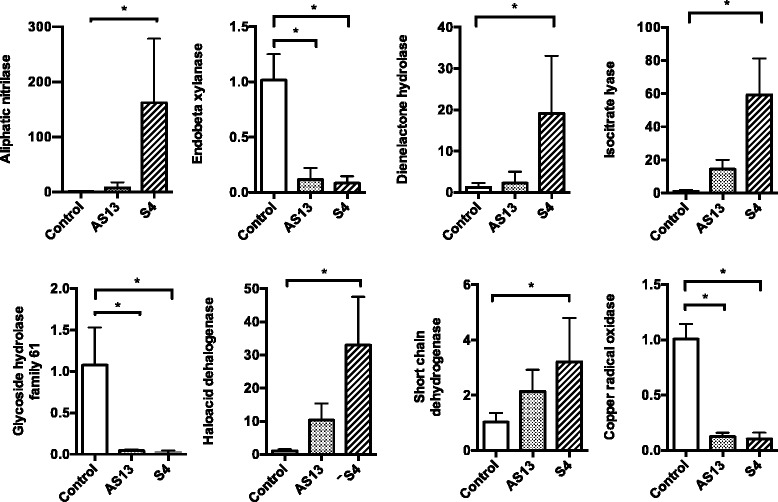
Expression profiles of selected *R. solani* genes during interaction with S4 *Serratia proteamaculans* and AS13 *Serratia plymuthica*. Relative expression levels in relation to Histone H3 expression are calculated from Ct values according to the 2 ^–ΔΔCt^ method. Error bars represent standard deviations based on 3 biological replicates. Asterisks indicate statistically significant differences compared with expression in the absence of bacteria (control) according to Fisher’s test (P ≤ 0.05)

To validate the RNA-seq data, we used quantitative real-time PCR (qRT-PCR) to examine the pattern of gene expression of eight highly up- or down- regulated DEGs. These genes encoded aliphatic nitrilase, endobeta xylanase, dienelactone hydrolase, isocitrate lyase, glycoside hydrolase family 61, haloacid dehalogenase, short chain dehydrogenase and copper radical oxidase. The trend of up- or down- regulation observed in the RNA-seq experiment was validated for all the genes and statistically significant differences were observed between control and both treatments for three genes. For the remaining five genes, statistically significant differences were observed between control and the treatment with S4 (Fig. 6).

## Discussion

 The results presented in this study support our hypothesis, that when *R. solani* is confronted with *S. proteamaculans* S4 and *S. plymuthica* AS13, it regulates expression of genes associated with general stress responses common to both bacteria, that involve both primary and secondary metabolism. We interpret the genes in the main Gene Ontology categories oxidation-reduction, pathogenesis, threonine-type endopeptidase activity and cellular proteolysis as being involved in the following processes: a) arrested growth of the fungus and changes in hyphal morphology, b) defence against bacterial stress through the production of antioxidants, xenobiotic degradation and environmental alterations and c) attack involving toxin production and oxidative stress. Although no significant general differences between the fungal responses to the two species of bacteria were found, differences in specific KEGG metabolic pathways and individual genes were evident between the two bacterial treatments.

### a) Changes in growth and hyphal morphology

Arrested growth of the fungal hyphae was evident at an early stage in our *in-vitro* systems indicating that cell division and regulation of genetic information processing was under repression. Indeed, genes related to transcription, translation, DNA repair and replication were downregulated in the presence of S4 and AS13. Changes in hyphal growth and morphology of the pathogen in response to the bacteria are indicative of stress responses by the fungus. These appeared to involve genes related to the regulation of cell walls and cell membrane functions. The use of the biological control bacterium *Pseudomonas fluorescens* DR54 induced similar morphological changes in *R. solani* hyphae [66].

The fungal cell wall is a dynamic structure responsible for protection of the cell from changes in osmotic and environmental stresses and is the first barrier that needs to be overcome to achieve invasion of host cells. Fungal cell walls are primarily composed of chitin, glucans, mannans and glycoproteins [10]. Consequently, differential expression of cell-wall modification genes is expected when fungi are exposed to stress-related conditions. In our study, a chitinase gene, a member of the glycoside hydrolase family 18 protein involved in chitin catabolism, as well as genes encoding for chitin deacetylase and glucanase, were found to be repressed in the presence of both of the bacteria. This is consistent with our microscopic observation of thickened fungal cell walls when *R. solani* was challenged with the bacteria.

 Depletion of ergosterol in fungal membrane disrupts the structure and many functions, leading to inhibition of fungal growth [61]. Additionally, regulation of membrane permeability is one of the principal mechanisms for conferring protection against oxidative stress [40, 63]. In our study, the ergosterol biosynthesis genes ERG2 to ERG6 were all significantly downregulated during interaction with S4, with fold values ranging between −3 to −6. In contrast, up-regulation of ergosterol related genes has been reported for the filamentous fungus *Aspergillus niger* when confronted with the mycophagous bacterium *Collimonas fungivorans*, probably as a mechanism for regulating membrane fluidity [42], or as conferring resistance to antifungal compounds such as amphotericin B [25].

 ABC transporters and other efflux pumps are exploited for active export of toxins out of the cell in order to reduce toxin concentration at the target sites and prevention of accumulation of harmful secondary metabolites produced during pathogenesis [19]. This has been reported for the pathogen *Botrytis cinerea* in presence of the antimicrobial compounds 2,4-DAPG and pyrrolnitrin produced by biological control bacteria of the genus *Pseudomonas* [59]. In a previous study of fungal interactions with *Serratia* sp. transcipts for production of pyrrolnitrin were upregulated in *S. plymuthica* AS13 but not in *S. proteamaculans* S4 [47]. Interestingly in the present study one gene encoding an ABC transporter was upregulated only in the presence of AS13 suggesting its role in protection from bacterial metabolites.

Plant cell-wall degrading enzymes can be downregulated in the presence of antagonistic bacteria suggesting that pectin, cellulose and glucose might have become depleted in the growth medium. In the presence of S4 one gene encoding polysaccharide monooxygenase was downregulated. One gene related to pectinesterase activity was also found to be downregulated in the presence of S4 and two genes encoding glucan 1,3-beta-glucosidases as well in presence of both S4 and AS13. Two cellulase genes, members of the glycoside hydrolase family 45, were downregulated in both the S4 and AS13 treatments. These observations suggest that bacterial antagonists may induce nutrient stress in the plant pathogen.

Organisms under stress conditions have been shown to increase fatty-acid degradation, upregulate genes involved in the glyoxylate cycle and store glycogen and trehalose [18, 29]. In our study four genes related to fatty acid degradation, four genes related to pyruvate metabolism and one gene related to fatty acid metabolism were significantly upregulated in both of the *Serratia* treatments, suggesting that under stress these compounds could potentially be used as carbon sources through gluconeogenesis. Acetate-ligase is a key enzyme related to pyruvate metabolism and gluconeogenesis and was induced in the presence of *Serratia*.

The key enzymes of the glyoxylate pathway (isocitrate lyase and malate synthase) were highly expressed in the presence of both *Serratia* bacteria. In this pathway, the enzyme isocitrate lyase cleaves isocitrate into succinate and glyoxylate and subsequently glyoxylate is converted to malate by the enzyme malate synthase [4]. A deletion strain of the isocitrate lyase gene in *Trichoderma atroviride* showed reduced antagonistic ability against *Botrytis cinerea* and induction of systemic resistance in *Arabidopsis thaliana,* demonstrating the importance of the glyoxylate cycle in growth, stress tolerance and antagonism [18].

Glycogen and trehalose are two glucose compounds that have been demonstrated to be used by fungi, playing crucial roles in metabolic adaptation including stress protection and energy storage and putatively in progression of cell division [29]. Enzymes for the metabolic synthesis of both compounds were upregulated in the presence of AS13, whereas presence of S4 led to the induction of trehalose biosynthesis, that has been associated with stress tolerance [22].

Genes involved in nitrogen metabolism (nitrate reductase, nitrite reductase, nitrate/nitrite transporter) were upregulated only in the treatment with S4. Increased expression levels of nitrogen metabolism genes were found in S4 bacteria only, but not in AS13 [47].

Protein kinases have roles in all aspects of regulation and signal transduction and are responsible for protein phosphorylation. Protein kinases such as serine/theorine, MAPKs, adenylsulfate, ribosylnicotinamide were up-regulated in both bacterial treatments. Two-component regulatory systems serve as a basic stimulus response coupling mechanism, allowing organisms to sense and respond to changes in environmental conditions [64] and enzymes involved in this signal transduction system were induced (alkaline phosphatases).

### b) Defence

Plant pathogenic fungi have evolved a wide array of mechanisms to defend themselves during stress and competition, either in an attempt to ameliorate the effects of toxic compounds, or to outcompete other microorganisms in terms of scavenging nutrients, occupying ecological niches and altering the environment [19]. Our data indicate that *R. solani* can defend itself against oxidative stress caused during confrontation with *Serratia* S4 and AS13 through two mechanisms: 1) antioxidant production and degradation of xenobiotics and 2) environmental alteration.

1. *Antioxidant production and xenobiotic degradation*

A common defence mechanism of all living organisms is related to the production of antioxidants that remove free radical intermediates and inhibit other active oxidants and it is likely that *R. solani* also attempts to increase resistance to oxidative stress caused by both bacteria. Almost 20 (26 during challenge with S4 and 16 during challenge with AS13) transcripts related to oxidoreductase activity were upregulated in both treatments and in terms of defence such genes could be used for xenobiotic degradation and serve as antioxidants. One such example is glutathione metabolism where six and three genes encoding for glutathione-S-transferase (GST) proteins were upregulated between 8 and 100 times in the presence of S4 and AS13 respectively.

Transaminases and pyridoxal-5-phosphatases are enzymes implicated in the biosynthesis of Vitamin B6 and the genes encoding the production of these enzymes were upregulated 2–6 times in response to interaction with S4. Pyridoxal reductase is essential for the biosynthesis of vitamin B6, which has been shown to act as an antioxidant and alleviator of reactive-oxygen-species (ROS) in stressed fungi [8]. It has been shown that when *R. solani* is confronted with the mycoparasite *Stachybotrys elegans*, pyridoxal reductase expression is induced, probably as a defence response [12].

Epoxide hydrolases were initially reported as enzymes involved in detoxification of different epoxides, including 1,3-butadiene oxide, styrene oxide, polycyclic aromatic hydrocarbon benzo(a)pyrene-4,5-oxide [3, 23, 72]. The genome of *S. plymuthica* AS13 contains genes for acetoin reductase involved in conversion of acetoin to 2,3-butanediol, as well as 2,3-butanediol reductase involved in catabolism of 2,3 butanediol [47]. The molecule 2,3-butanediol can be dehydrated to 1,3-butadiene [65]. During confrontation of *R. solani* with S4 and AS13, two and one genes respectively, encoding for microsomal epoxide hydrolase (EC 3.3.2.9) were highly induced between 4 and 50 times.

 Nitrilases catalyze the hydrolysis of nitrile compounds to carboxylic acid and ammonia. In plant-microbe interactions, they are implicated in hormone synthesis, nitrogen utilization, catabolism of cyanogenic glycosides and glucosinolates and detoxification of nitriles and cyanide [31, 34, 54]. Bacterial nitriles can be formed during detoxification of endogenous and exogenous cyanide produced during cyanogenesis [32] and *Serratia* AS13 can produce HCN. In addition, the genomes of both *Serratia* S4 and AS13 contain genes that catalyse reactions involved in indole-3-acetic acid (IAA) biosynthesis [47] and nitrilases have been implicated in the conversion of IAA precursors to IAA [53, 55]. In the presence of both bacteria, the enzyme aliphatic nitrilase was highly upregulated between 30 and 100 times. Similar patterns of highly upregulated expression of nitrilase genes have been reported for *Aspergillus niger* during confrontation with the bacterium *Collimonas* [42].

Halogenated compounds can be synthesized in nature by a wide range of microorganisms, some of which have antimicrobial properties [27]. It is known that fungi can degrade antibiotics produced by competing microorganisms and 50 times upregulation of the gene encoding for haloacid dehalogenase could be implicated in the degradation of the antibiotic pyrrolnitrin produced by *Serratia* S4 and AS13 [47, 48].

Fungal laccases have multiple roles, including morphogenesis, degradation of lignin, stress defence and plant/pathogen interaction [67]. In our study, four genes encoding for laccase multicopper benzenediol:oxygen oxidorectuctase were upregulated in response to both bacteria from 1.5 to 8 times. In another study, different strains of the biological control bacterium *Pseudomonas fluorescens* induced elevated laccase production in *R. solani* interpreted as triggering of calcium and heat/shock signalling pathways in response to bacterial antifungal metabolites [15].

2) *Environmental alterations*

Some species of fungi are capable of gaining a competitive advantage over other microorganisms by acidifying their environment. Increased production of oxalate by *R. solani* in response to *P. fluorescens* [46] and by *Aspergillus niger* in response to the bacterium *Collimonas* [42] has been shown. When *R. solani* was challenged with *Serratia* S4, 2 genes encoding oxalate decarboxylase were overexpressed 6–15 times. The underlying reasons for this change in our experiment are not clear but fungal oxalate decarboxylases are considered to play a major role in the prevention of high intracellular levels of oxalic acid as well as to the decomposition of extracellular oxalic acid [41, 44].

### c) Attack

Toxin production was induced in both treatments, volvatoxin a2 precursor in treatment with S4 and partial delta-endotoxin in treatment with AS13. Both toxins are members of the Endotoxin CytB protein family, having insecticidal properties and produced by the bacterium *Bacillus thuringiensis* [11]. It has been reported that similar proteins can be found in other pathogenic fungi and bacteria suggesting a potential role of these proteins in the virulence of these microorganisms [62]. Interestingly, it has been recently shown that when *R. solani* AG3 was co-cultured with the soil-inhabiting bacterial antagonist *Bacillus subtilis* and with the mycoparasite *Stachybotrys elegans*, *RSENDO1* gene encoding a delta-endotoxin CytB gene was downregulated in contrast to the upregulation shown in our study [13]. A gene containing the ricin b-like lectin domain involved in the production of the highly toxic legume lectin ricin was also highly upregulated almost 16 times in both treatments.

The survival, response and adaptation of cells to environmental changes is strongly dependent on processes involving proteolysis [43] and proteolytic enzymes of numerous phytopathogenic fungi are potential pathogenicity factors. In this respect it is interesting that genes encoding for proteases were induced in both of the treatments. In the S4 treatment, six such genes encoding the metalloprotease deuterolysin were upregulated and could possibly be a defence reaction of the fungus. The metalloprotease serralysin was also found upregulated in S4 and AS13 *Serratia* bacteria interacting with *R. solani* in an earlier study [47].

## Conclusions

RNA sequencing technology allowed the identification of a large number of genes in the phytopathogenic fungus *R. solani* required for survival and defence in the presence of plant-associated bacteria *S. plymuthica* (AS13) and *S. proteamaculans* (S4). A major shift in gene expression was observed with a simultaneous alteration of primary metabolic processes, activation of defence and attack mechanisms and changes in hyphal morphology. Most genes were regulated in a similar way in the presence of both bacterial strains, but there were also some strain-specific responses. Most of these were related to the presence of *S. proteamaculans* S4, a finding that is consistent with its apparently higher degree of antagonism against *R. solani.*

 Obviously, the controlled environment used in the above study lacks the complexity of real-life soil and rhizosphere habitats, but nevertheless the results provide an insight into the functional basis of the responses of a fungal pathogen to two closely related antagonistic bacteria. The differentially expressed genes identified with our *in-vitro* experimental approach provide a context within which to perform further analyses of a) this fungal isolate to other species and strains of bacteria, b) other members of the *Rhizoctonia* species complex to *Serratia* bacteria, and c) *R. solani* AG3 gene expression in more natural soil systems. The long term aim of these studies is to provide a more detailed understanding of the *in-situ* competitive interactions between bacteria and fungi, that in turn will permit successful application of biological control strategies within sustainable agriculture.

## Methods

### *In-vitro* dual culture assays

*In-vitro* bacterial-fungal dual-culture assays were established in 9 cm diameter Petri dish systems containing half strength Potato Dextrose Agar (PDA) medium. A 5-mm diam plug taken from the edge of an actively growing colony of *R. solani* Rhs1AP was inoculated in the centre of each Petri dish. Fresh cells of *S. proteamaculans* S4 or *S. plymuthica* AS13 [50, 51] were streaked in 3-cm length parallel line 3-cm on each side of the fungal plug. Control treatments with fungus only were also set up. The *R. solani* isolate used was the Rhs1AP AG-3.

Fungal hyphae from control and from challenged with *Serratia* treatments was stained with the vital stain phenossaffranin (199648 Sigma-Aldrich) and photographs were taken using a Zeiss Axioplan microscope (Thornwood, NY) equipped with Leica application suite version 3.6.0 at 40x and 100x magnification.

### RNA-extraction

Total fungal RNA was extracted 72 hours post-inoculation using the RNeasy Plant Mini Kit (Qiagen). For treatments where the fungus was challenged with the bacteria, RNA was extracted from the zone of interaction (1 cm). The peripheral fungal zone was used for RNA isolation in the controls containing only the fungus. RNA integrity was analysed after DNase I treatment (Fermentas, St. Leon-Rot, Germany) by electrophoresis on an Agilent Bioanalyzer using the RNA 6000 Nano kit (Agilent Technologies, Santa Clara. CA) and finally 5000 ng – 1ug of total RNA were sent to SciLife Lab, Uppsala and subjected to Illumina® TruSeq sequencing.

#### Quantitative Real-Time PCR validation

Verification of expression profiles of the Illumina sequencing data was carried out by qRT-PCR analysis. Total RNA was extracted as described above. To generate first strand cDNA, reverse transcription was carried out with 180 ng of total RNA in a 25 μl reaction using iScript^TM^ cDNA Synthesis Kit (BioRad, Hercules, CA). Transcript levels were quantified by RT-qPCR in an iQ5 qPCR System (BioRad, Hercules, CA). Each 20 μl reaction contained 2 μl of 2-fold diluted cDNA template, 400nM of each primer and 10 μl of SsoFast™ EvaGreen® Supermix (BioRad, Hercules, CA). The amplification program consisted of: 98 °C for 2 min, 40 cycles of 98 °C for 5 s and 60 °C for 10 s. Melt curve analysis was conducted to confirm a single amplification product. Primer amplification efficiency was deducted from amplification of standard curves using dilution series of *R. solani* cDNA. Expression of eight genes was normalized by Histone-3 [12]. The choice of the reference gene was based on its lowest coefficient of variation of 2.32 %, with standard deviation of 0.60 % when compared with GAPDH and alpha-tubulin genes, using the Bestkeeper software [57]. Relative expression values were calculated according to the 2^–ΔΔCt^ [38]. Transcript levels were quantified in three biological replicates, each based on three technical replicates. Primers used for RT-qPCR were designed towards sequences of *R. solani* genes to amplify amplicons ranging from 80–200 bp using the Primer 3 software [35, 69], (Table 4). Analysis of variance (ANOVA) was conducted using a General Linear Model implemented in SPSS ver. 21 (IBM, Armonk, NY). Pairwise comparisons were made using Fisher’s method at the 95 % significance level.

**Table 4:** Primers used for qRT-PCR validation of RNAseq data

Gene Id	Putative function	Forward primer	Reverse primer	Amplicon bp
1.t002520	aliphatic nitrilase	GCGCGAACTTTGTGTCGATT	AACGGCATTGACTTTGGGAG	100
15.t000002	endobeta xylanase	GCGCGAACTTTGTGTCGATT	AACGGCATTGACTTTGGGAG	122
1.t001237	dienelactone hydrolase	CAGGAGACAATGCAGCTTGT	ATCACAACGACGATGGCATG	139
4.t000274	isocitrate lyase	GGGCTTTGTATGGCAGTTCA	TAGTCGGCACCAGACCATTT	180
1.t002315	glycoside hydrolase family 61 protein	CCTGGCACCGACAAAGTTT	CGTGACGCATGATGTACTGG	150
6.t000086	haloalkanoic acid dehalogenase	GCGAGAGAAAATGTGACTATTGG	ACTCTGTCTCTGCTGCATCT	97
1.t000612	short chain dehydrogenase	TCCAGAGATCGATTGCCTCC	AAGGTGAACGAGGCCAGTAA	124
2.t000524	copper radical oxidase	TGTTGCCTCTCTTCTTCCGT	CAGTGTATGTCGGCCTTTCG	146

### Bioinformatic analysis

#### Read trimming and mapping of Illumina reads

Illumina adaptor sequences and low quality bases from reads were removed using the software Nesoni (http://www.vicbioinformatics.com/software.nesoni.shtml), using a quality cutoff of 20 and discarding reads shorter than 75 bp. Bioinformatic analysis of trimmed RNA-seq reads was conducted using the Tuxedo Suite [68]. More precisely, RNA-seq reads obtained from three biological replicates for each treatment (*R. solani* control, *R. solani* + S4, *R. solani* + AS13) were mapped to the *R. solani* AG-3 Rhs1AP reference genome (http://www.ncbi.nlm.nih.gov/bioproject/73133) using firstly the fast read aligner Bowtie2 (2.1.0), including the parameters I and X both in default values [36] to calculate the mean inner distance between the mate pairs, followed by the spliced read aligner TopHat2 including the mean inner distance (−r) and the standard deviation for the distribution of the inner distances as calculated by Bowtie2, the -read-realign-edit-dist parameter set to 0 and the –g option set to 1 (2.0.9).

#### Abundance estimation and Differential expression analysis

Cufflinks (2.1.1) assembled transcripts and quantified transcript abundance in terms of fragments per kilobase of exon per million mapped fragments (FPKM). Parameters activated during the run were –u, −F set to 0.1, −j set to 0.1 as well as –N allowing for upper quartile normalization. Cuffmerge was subsequently used to merge the input assemblies from the multiple RNA-seq libraries and the output files were used as input to Cuffdiff. Cuffdiff was used to test for statistically significant differences in transcript expression between 2 comparison pairs using multi-read correction –u and –F (minimum isoform fraction) to default value. Differentially expressed genes (DEGs) were identified using the two criteria a) log_2_ fold-change > 3 and b) q-value (false discovery rate (FDR)) < 0.05.

#### Functional classification and annotation of DEGs

Sequence similarity was performed using the BLASTx algorithm at statistical significance threshold 1.0E-6. Functional categories were assigned to the differentially expressed genes according to the Gene Ontology (GO) system using Blast2GO, enabling the integrated Interproscan and ANNEX functions for improved annotations [14]. Enrichment of GO terms in biological process, molecular function and cellular component categories was evaluated by Fisher’s exact test with a FDR threshold of 5 %. In addition, protein sequence fasta files were submitted in KAAS (KEGG automatic annotation server) [45] and KEGG orthology assignments were obtained as well based on bi-directional best-hit of BLAST to a threshold of 60.

### Availability of supporting data

The data discussed in this publication have been deposited in NCBI’s Gene Expression Omnibus [21] and are accessible through GEO Series accession number GSE66652 (Short Read Archive accession number SRP055989) (http://www.ncbi.nlm.nih.gov/geo/query/acc.cgi?acc=GSE66652).

## Additional file

### GO classification

#### GO classification during challenge with S4 and AS13 *Serratia*

In the Molecular Function classification, the up-regulated transcripts during confrontation with both S4 and AS13 bacterial strains were mainly categorized into oxidoreductase, hydrolase, lyase, transferase and transporter activity, some binding child categories such as protein, nucleotide, ion, lipid and carbohydrate as well as in molecular signal transducer activity. Even though no significant difference was observed, terms such as metal cluster binding, nucleoside-phosphatase regulator and selenium binding were present only in the treatment with S4. There was also a pattern of greater induction of hydrolases when AS13 was present. The downregulated transcripts were mainly grouped into hydrolase, oxidoreductase and binding, especially nucleic acid, protein and ion binding. Again there was no significant difference, however in S4 treatment the main repressed functions belonged to binding (special “child” terms), whereas in AS13 treatment oxidoreductase, hydrolase and transporter activities were mainly inhibited (Additional file 12: Figure S10).

Examination of Biological Process GO terms suggested that genes involved in primary metabolism such as protein, carbon, nitrogen, carbohydrate and nucleic acid, regulation of biological and cellular processes, catabolism, oxidation reduction processes, transport, secondary compound metabolism and response to stimulus were upregulated in confrontation with both bacteria. More terms related to biosynthetic processes and transport were found in S4 treatment, whereas the AS13 treatment was enriched in terms involved in metabolism (primary, nitrogen compound metabolic process, cellular metabolic process and catabolism). Primary and cellular metabolic processes, oxidation reduction, catabolism, biosynthesis and biological and cellular regulation were the main categories downregulated. On one hand, nitrogen and alcohol compound metabolism, catabolism, organelle organization, ribonucleoprotein complex biogenesis and DNA packaging were downregulated to greater extent in the presence of S4 compared to AS13. On the other hand, oxidation reduction, trensmembrane transport and pigment metabolism were the prominent downregulated terms in AS13 inoculated treatments (Additional file 13: Figure S11).

#### Comparison of GO terms between induced and repressed genes

Comparison of more general molecular function GO functional categories in the differentially expressed genes between the induced (log_2_FPKM > 0) and repressed (log_2_FPKM < 0) genes of the treatment with S4 revealed that transferase activity as well as lyase, deaminase, transmembrane transporter and nucleotide and cofactor binding were enriched in the up-regulated list, whereas nucleic acid and tetrapyrrole binding were enriched in the down-regulated list (Additional file 14: Figure S12). Biological process terms related to primary metabolism eg. carbon utilization, organophosphate and secondary metabolism, electron transport, cell communication, cellular homeostasis and interspecies interactions between organisms were only present in the up-regulated list. On the other hand, DNA packaging, cell division and organization, pigment metabolism and protein complex biogenesis were only present in the list of down-regulated genes (Additional file 15: Figure S13).

When comparing Molecular Function terms of the treatment with AS13, transferase, signal transducer activity, lyase, isomerase, deaminase and child binding terms (cofactor, ion, vitamin, lipid, protein, nucleoside and nucleotide) were prominent in the list of upregulated genes. Conversely, oxidoreductase, hydrolase, transporter activity and tetrapyrrole, carbohydrate, nucleic acid and pattern binding were more abundant in the downregulated list (Additional file 16: Figure S14). Similarly to the S4 treatment, primary and cellular metabolism, organophosphate and secondary metabolism and catabolism were biological process terms found enriched, whereas nitrogen compound metabolism, oxidation reduction, pigment, transporter activity, and response to stress were found enriched in the downregulated list (Additional file 17: Figure S15).

In all of the comparisons, it was found that genes involved in signal transducer activity were upregulated. Catabolic enzymes, not only hydrolases but also lyases and deaminases were present in both up- and down- regulated gene lists. Also, nucleic acid, nucleotide and nucleoside binding terms were found abundant in our study, both induced and repressed.

#### Comparison with the reference genome of *R. solani* RhS1AP for DEGs log_2_(3) or more

For the *R. solani* genes that exhibited differential expression patterns log_2_(3) or more during the treatment with S4, 22 upregulated enriched gene groups were identified both over- and under- represented. Over-represented biological process GO terms included, GO:0032787 (monocarboxylic acid metabolic process) and GO:0042967 (acyl-carrier-protein biosynthetic process) whereas molecular function terms were GO:0003824 (catalytic activity), GO:0016746 (transferase activity, transferring acyl groups), GO:0008080 (N-acetyltransferase activity) and GO:0016410 (N-acyltransferase activity). On the other hand, under-represented GOs basically involved cellular component terms GO:0005623 (cell), GO:0005622 (intracellular), GO:0032991 (macromolecular complex), as well as some molecular function and biological process terms GO:0003676 (nucleic acid binding), GO:0010467 (gene expression), respectively (Additional file 4: Table S1). However, when testing the rest of the datasets (S4 down-regulated, AS13 up-regulated, AS13 down-regulated), no enriched terms could be identified at the threshold of False Discovery Rate (FDR) 0.05.

#### Comparison between the two bacterial treatments (S4 and AS13)

In order to broadly compare gene expression patterns a) between the two bacterial treatments (S4 and AS13), as well as b) between up- and down- regulated genes within the same treatment, functional categories were assigned to the DEGs according to Gene Ontology (GO) using Blast2GO [5, 14]. To enrich the category analysis for the up- and down- regulated genes between the two treatments, a Fisher’s Exact Test (FDR < 0.05) was performed but no statistically significant differences were found between the two bacterial treatments (S4 and AS13). Therefore, in order to investigate the functional categorization of *R. solani* genes when challenged with *Serratia* S4 and AS13 strains, the GO annotation obtained from Blast2GO was used and was visualized in Web Gene Ontology Annotation Plot (WEGO) [73].

## Additional files

Additional file 1: Figure S1. Box plot analysis of FPKM values distributions for the gene expression of *R. solani* triplicates grown in monoculture (C_0, C_1, C_2) and during challenge with a) S4 *Serratia proteamaculans* and b) AS13 *Serratia plymuthica* bacteria of the nine obtained RNA-seq libraries. (FPMK = fragments per kilobase of exon per million fragments mapped). Additional file 2: Figure S2. Principal Component Analysis (PCA) plots of the log_2_-transformed FPKM values for the gene expression of *R. solani* grown in monoculture (C) and during challenge with a) S4 *Serratia proteamaculans* and b) AS13 *Serratia plymuthica* bacteria. Dots represent expression values transformed into PC and arrows show how the given condition depends on the PC. (FPMK = fragments per kilobase of exon per million fragments mapped). Additional file 3: Figure S3. Volcano plots of *R. solani* genes that are differentially expressed when challenged with a) S4 *Serratia proteamaculans* and b) AS13 *Serratia plymuthica* bacteria compared to growth in monoculture. Red dots represent significant and blue dots non-significant, differential expression of up- and down- regulated genes. Additional file 4: Table S1. Diiferential Expression Data of genes being up- and down- regulated between control treatment and treatment challenged with *Serratia proteamaculans* S4. Additional file 5: Table S2. Diiferential Expression Data of genes being up- and down- regulated between control treatment and treatment challenged with *Serratia plymuthica* AS13. Additional file 6: Figure S4. Over- (**a.**) and under- (**b.**) represented Gene Ontology (GO) terms of *R. solani* genes up-regulated when confronted with S4 *Serratia proteamaculans*. Additional file 7: Figure S5. Over- (**a.**) and under- (**b.**) represented Gene Ontology (GO) terms of *R. solani* genes down-regulated when confronted with S4 *Serratia proteamaculans*. Additional file 8: Figure S6. Over- (**a.**) and under- (**b.**) represented Gene Ontology (GO) terms of *R. solani* genes up-regulated when confronted with AS13 *Serratia plymuthica*. Additional file 9: Figure S7. Over-represented Gene Ontology (GO) terms of *R. solani* genes down-regulated when confronted with AS13 *Serratia plymuthica*. Additional file 10: Figure S8. Over-represented Cellular Location terms from Gene Ontology (GO) of *R. solani* genes up- (**a.**) and down- (**b.**) regulated when confronted with S4 *Serratia proteamaculans.*
Additional file 11: Figure S9. Over-represented Cellular Location terms from Gene Ontology (GO) of *R. solani* genes up- (**a.**) and down- (**b.**) regulated when confronted with AS13 *Serratia plymuthica. *
Additional file 12: Figure S10. Comparison of Molecular Function annotations of *R. solani* genes between S4 *Serratia proteamaculans* and AS13 *Serratia plymuthica* treatments. a) Bar Chart of Gene Ontology (GO) annotations between S4-AS13 upregulated genes, b) Bar Chart of GO annotations between S4-AS13 downregulated genes (Values taken from WEGO). Additional file 13: Figure S11. Comparison of Biological Process annotations of *R. solani* genes between S4 *Serratia proteamaculans* and AS13 *Serratia plymuthica* treatments. a) Bar Chart of Gene Ontology (GO) annotations between S4-AS13 upregulated genes, b) Bar Chart of GO annotations between S4-AS13 downregulated genes (Values taken from WEGO). Additional file 14: Figure S12. Comparison of Molecular Function annotations of *R. solani* genes between the induced (log_2_FPKM > 0) and repressed (log_2_FPKM < 0) genes when challenged with S4 *Serratia proteamaculans*. (FPMK = fragments per kilobase of exon per million fragments mapped) Additional file 15: Figure S13. Comparison of Biological Process annotations of *R. solani* genes between the induced (log_2_FPKM > 0) and repressed (log_2_FPKM < 0) genes when challenged with S4 *Serratia proteamaculans*. (FPMK = fragments per kilobase of exon per million fragments mapped) Additional file 16: Figure S14. Comparison of Molecular Function annotations of *R. solani* genes between the induced (log_2_FPKM > 0) and repressed (log_2_FPKM < 0) genes when challenged with AS13 *Serratia plymuthica*. (FPMK = fragments per kilobase of exon per million fragments mapped). Additional file 17: Figure S15. Comparison of Biological Process annotations of *R. solani* genes between the induced (log_2_FPKM > 0) and repressed (log_2_FPKM < 0) genes when challenged with AS13 *Serratia plymuthica*. (FPMK = fragments per kilobase of exon per million fragments mapped).
