# Supplementary figures and images for: Transcriptomic changes in the plant pathogenic fungus Rhizoctonia solani AG-3 in response to the antagonistic bacteria Serratia proteamaculans and Serratia plymuthica
Source: BMC Genomics. 2015 Aug 22;16(1):630. doi: 10.1186/s12864-015-1758-z (PMC4546130; doi:10.1186/s12864-015-1758-z)

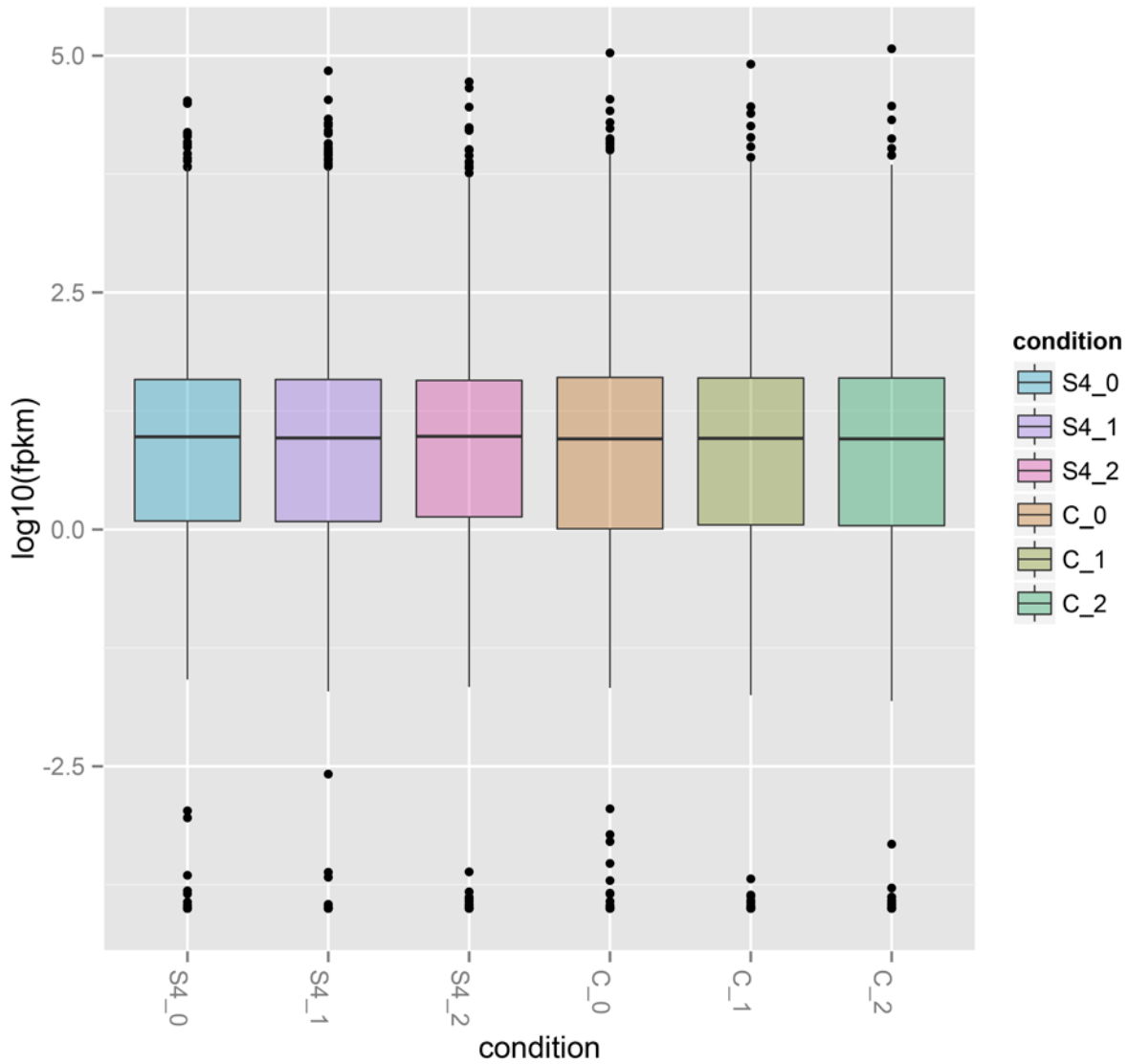

Supplement: Additional file 1: Figure S1. — Box plot analysis of FPKM values distributions for the gene expression of R. solani triplicates grown in monoculture (C_0, C_1, C_2) and during challenge with a) S4 Serratia proteamaculans and b) AS13 Serratia plymuthica bacteria of the nine obtained RNA-seq libraries. (FPMK = fragments per kilobase of exon per million fragments mapped). [file 12864_2015_1758_MOESM1_ESM.pdf]

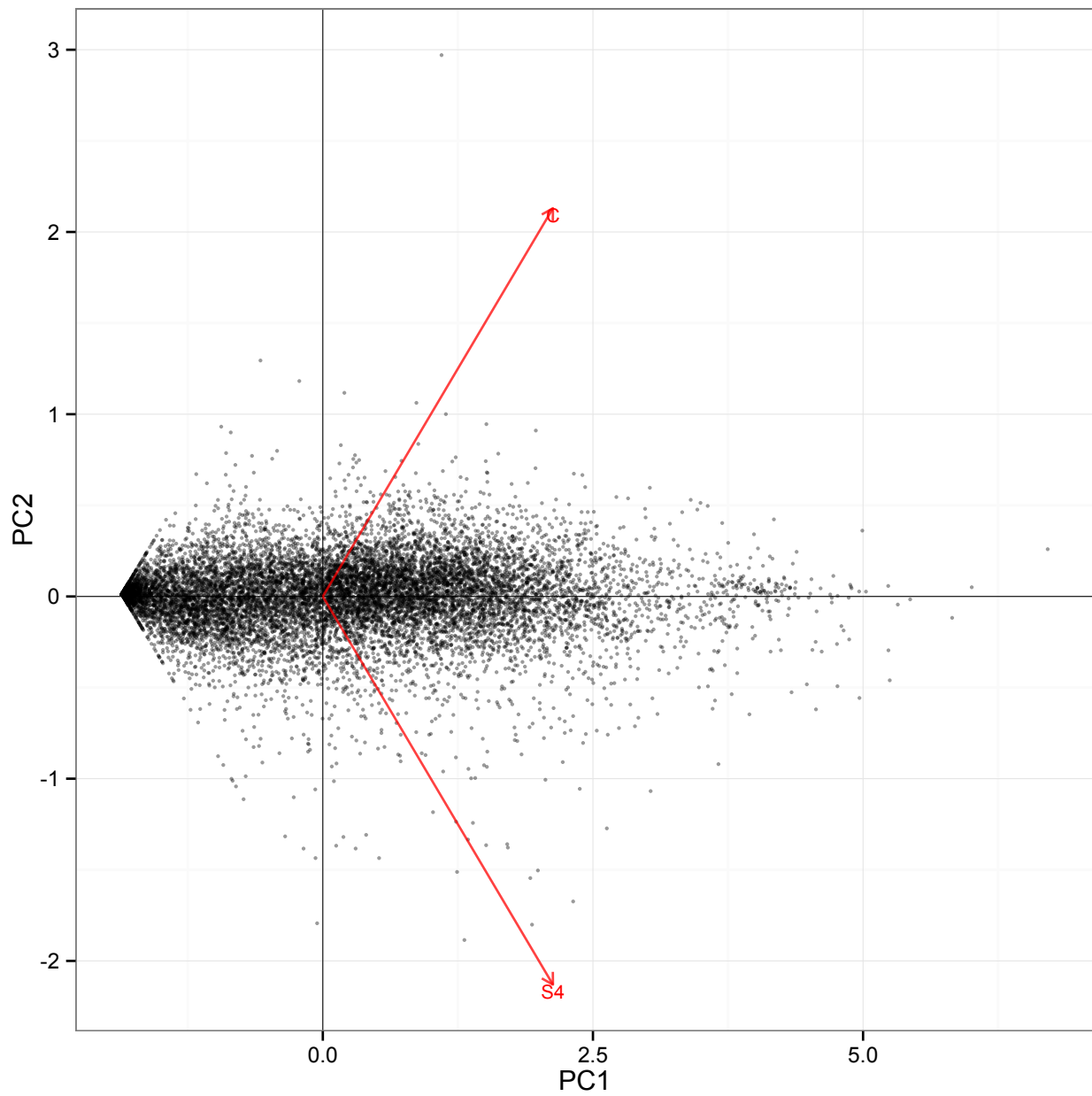

Supplement: Additional file 2: Figure S2. — Principal Component Analysis (PCA) plots of the log2-transformed FPKM values for the gene expression of R. solani grown in monoculture (C) and during challenge with a) S4 Serratia proteamaculans and b) AS13 Serratia plymuthica bacteria. Dots represent expression values transformed into PC and arrows show how the given condition depends on the PC. (FPMK = fragments per kilobase of exon per million fragments mapped). [file 12864_2015_1758_MOESM2_ESM.zip › Figure S2a.pdf]

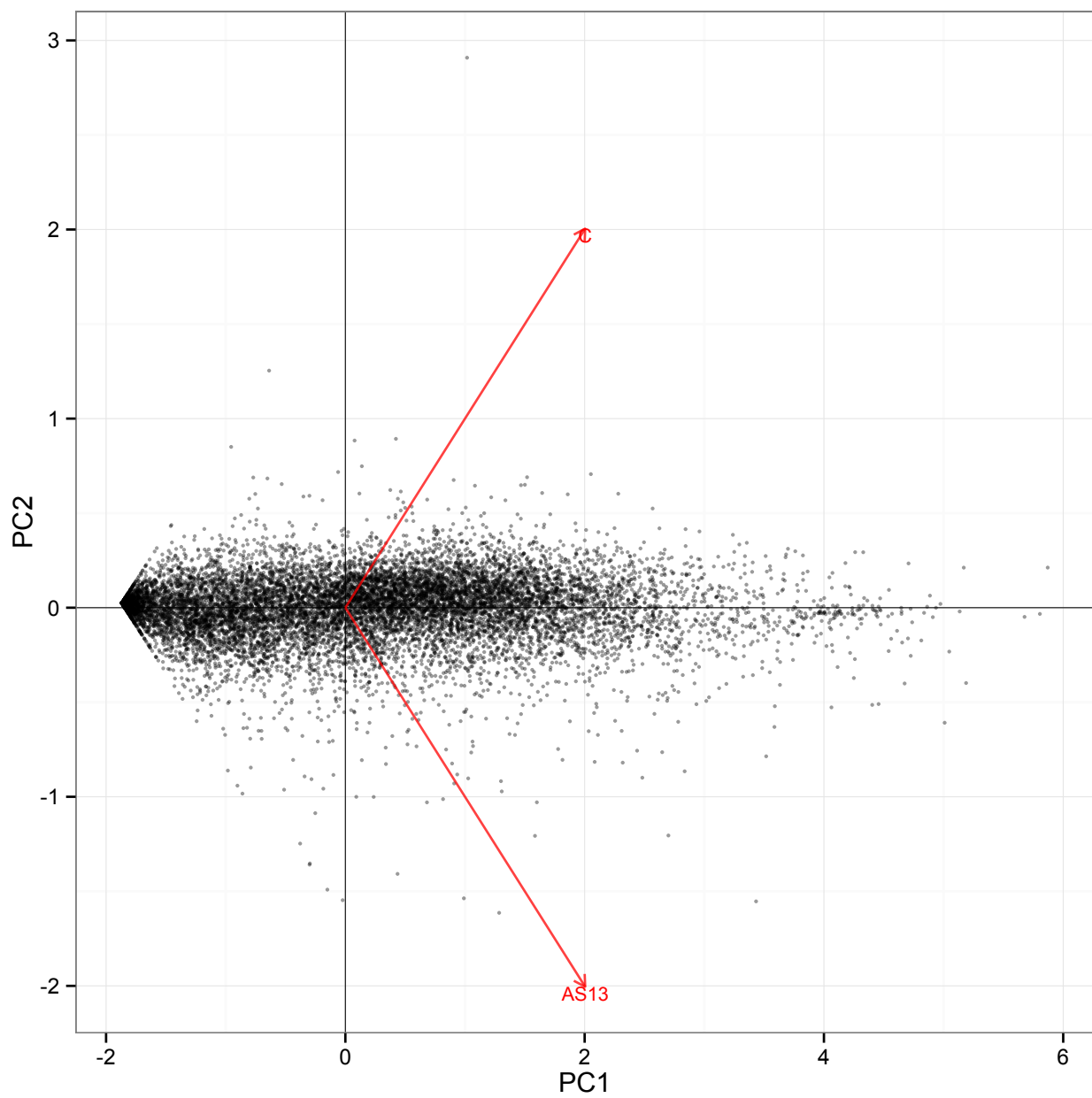

Supplement: Additional file 2: Figure S2. — Principal Component Analysis (PCA) plots of the log2-transformed FPKM values for the gene expression of R. solani grown in monoculture (C) and during challenge with a) S4 Serratia proteamaculans and b) AS13 Serratia plymuthica bacteria. Dots represent expression values transformed into PC and arrows show how the given condition depends on the PC. (FPMK = fragments per kilobase of exon per million fragments mapped). [file 12864_2015_1758_MOESM2_ESM.zip › Figure S2b.pdf]

a. S4 - *Serratia proteamaculans*

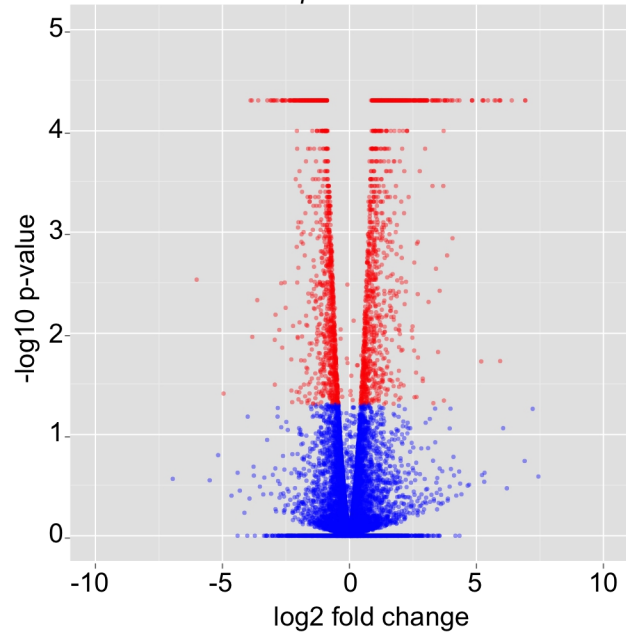

b. AS13 - *Serratia plymuthica*

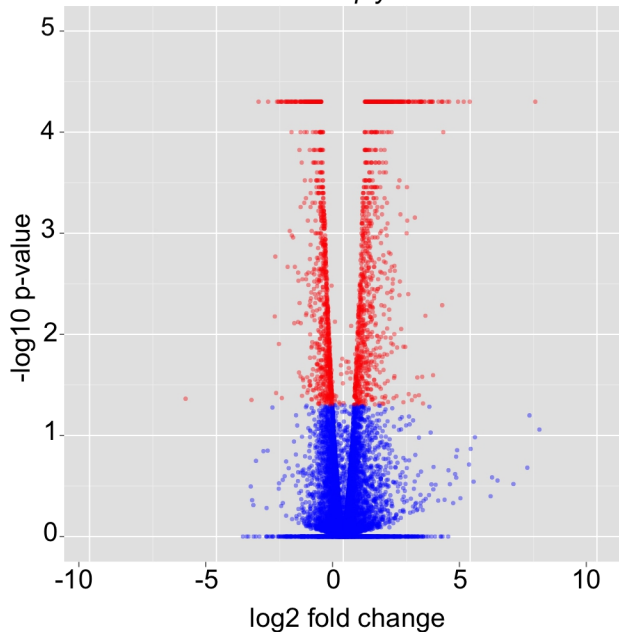

Supplement: Additional file 3: Figure S3. — Volcano plots of R. solani genes that are differentially expressed when challenged with a) S4 Serratia proteamaculans and b) AS13 Serratia plymuthica bacteria compared to growth in monoculture. Red dots represent significant and blue dots non-significant, differential expression of up- and down- regulated genes. [file 12864_2015_1758_MOESM3_ESM.pdf]

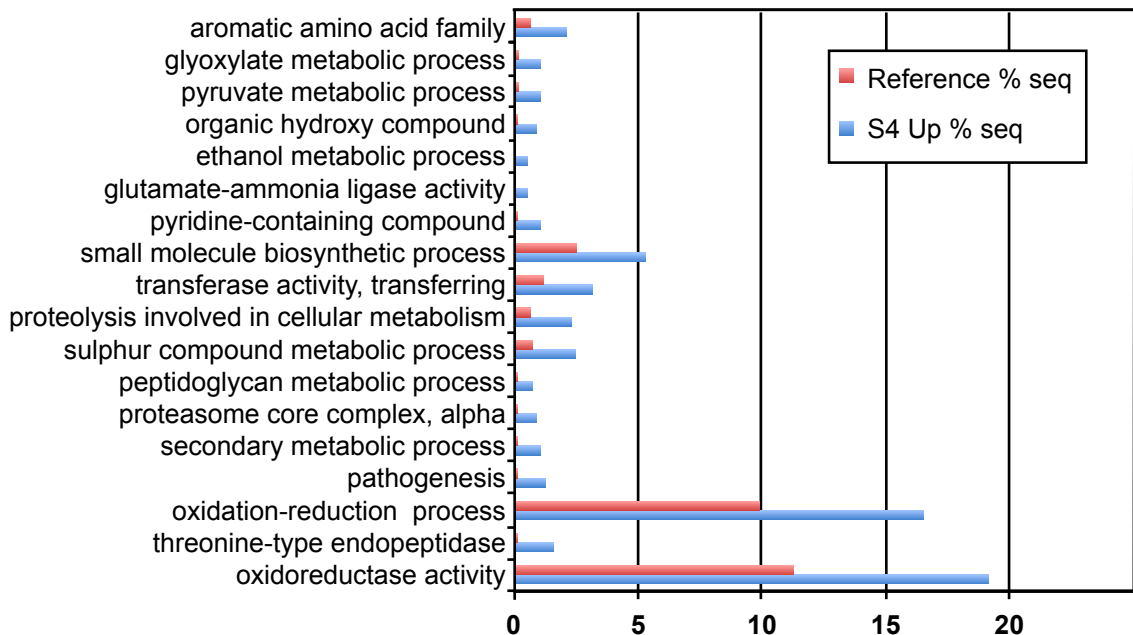

Supplement: Additional file 4: Table S1. — Diiferential Expression Data of genes being up- and down- regulated between control treatment and treatment challenged with Serratia proteamaculans S4. [file 12864_2015_1758_MOESM4_ESM.zip › Figure S4a.pdf]

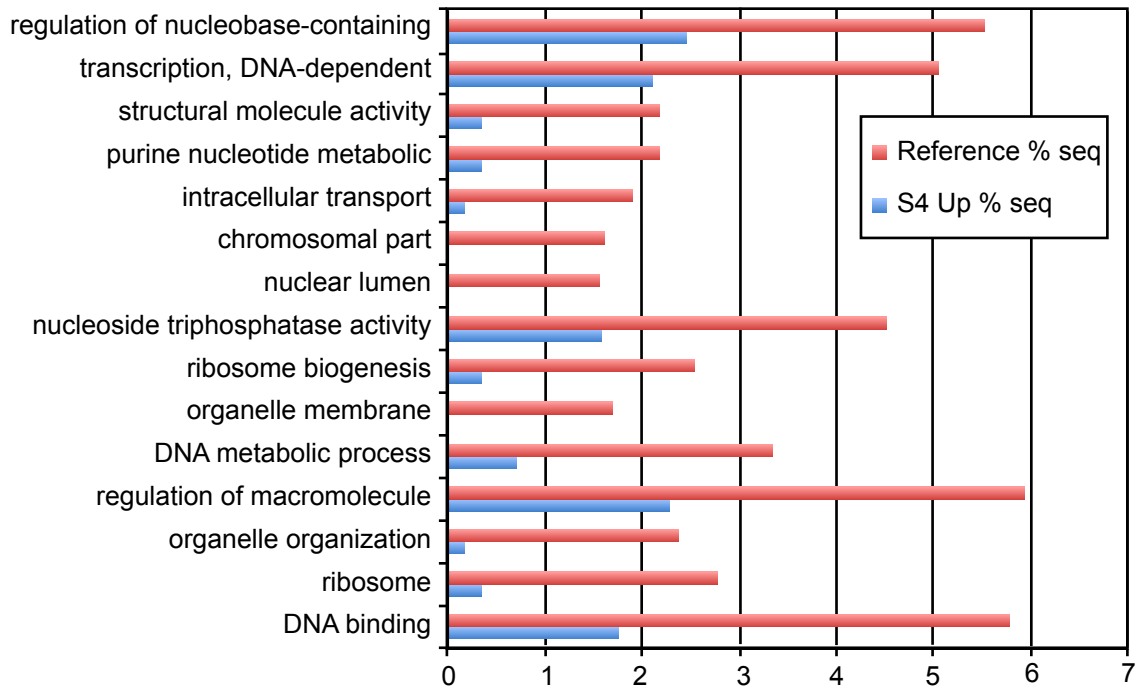

Supplement: Additional file 4: Table S1. — Diiferential Expression Data of genes being up- and down- regulated between control treatment and treatment challenged with Serratia proteamaculans S4. [file 12864_2015_1758_MOESM4_ESM.zip › Figure S4b.pdf]

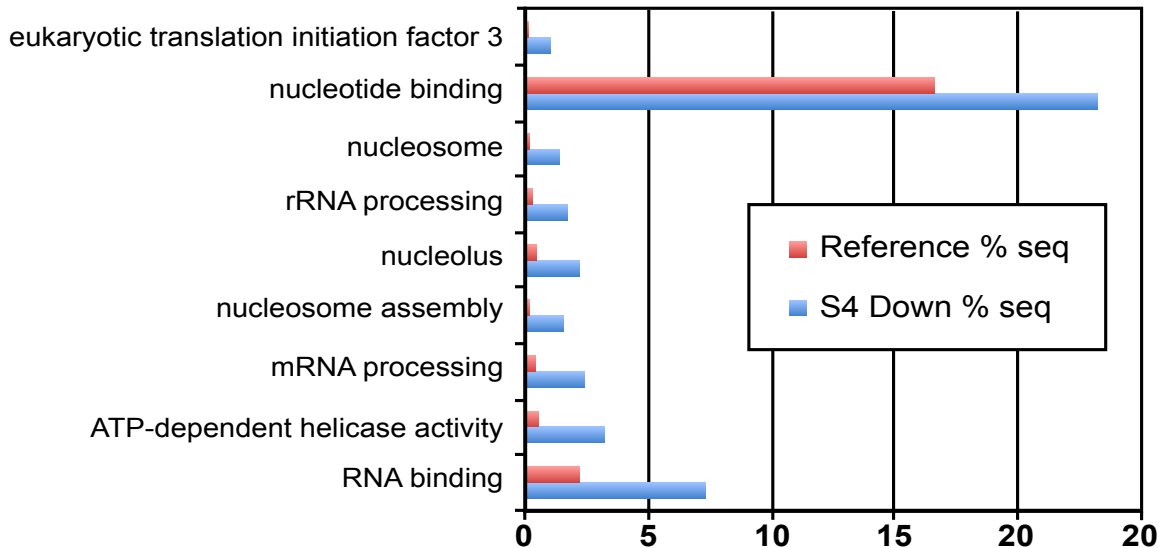

Supplement: Additional file 5: Table S2. — Diiferential Expression Data of genes being up- and down- regulated between control treatment and treatment challenged with Serratia plymuthica AS13. [file 12864_2015_1758_MOESM5_ESM.zip › Figure S5a.pdf]

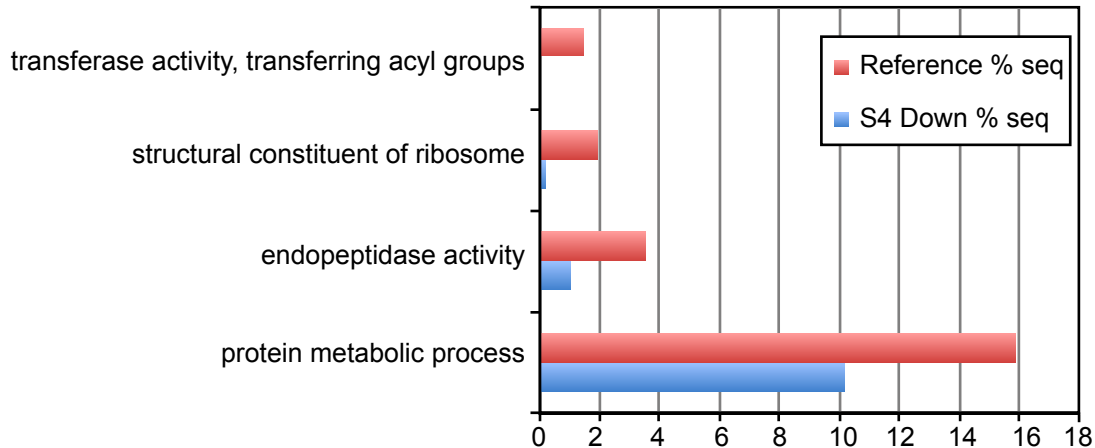

Supplement: Additional file 5: Table S2. — Diiferential Expression Data of genes being up- and down- regulated between control treatment and treatment challenged with Serratia plymuthica AS13. [file 12864_2015_1758_MOESM5_ESM.zip › Figure S5b.pdf]

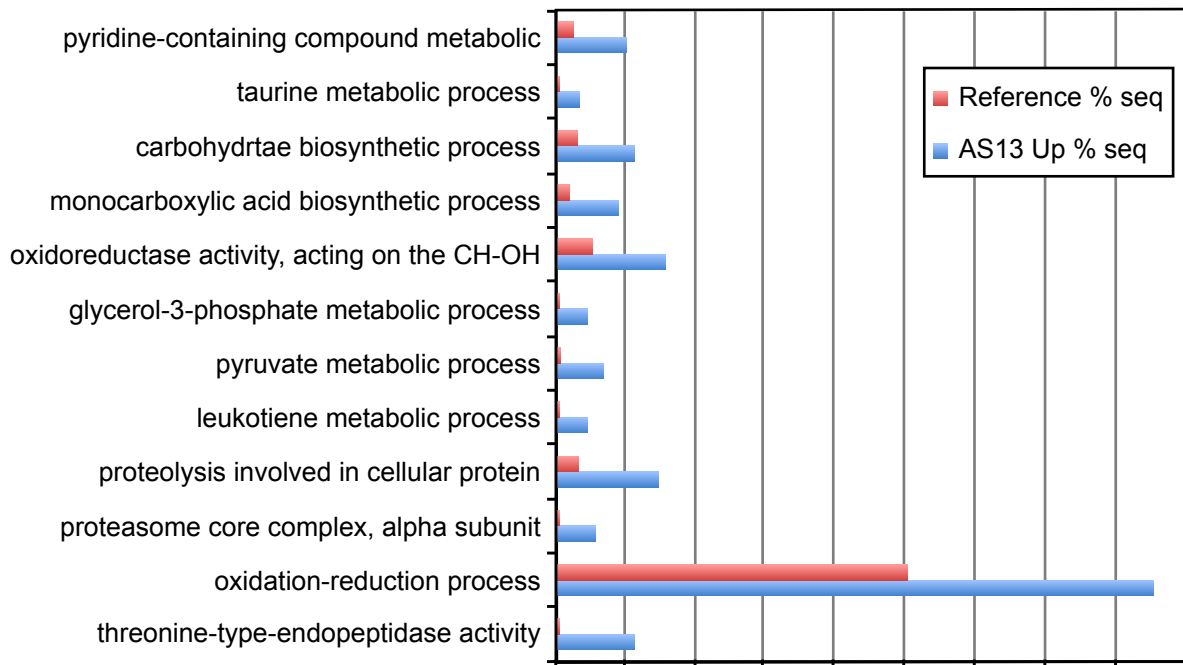

Supplement: Additional file 6: Figure S4. — Over- (a.) and under- (b.) represented Gene Ontology (GO) terms of R. solani genes up-regulated when confronted with S4 Serratia proteamaculans. [file 12864_2015_1758_MOESM6_ESM.zip › Figure S6a.pdf]

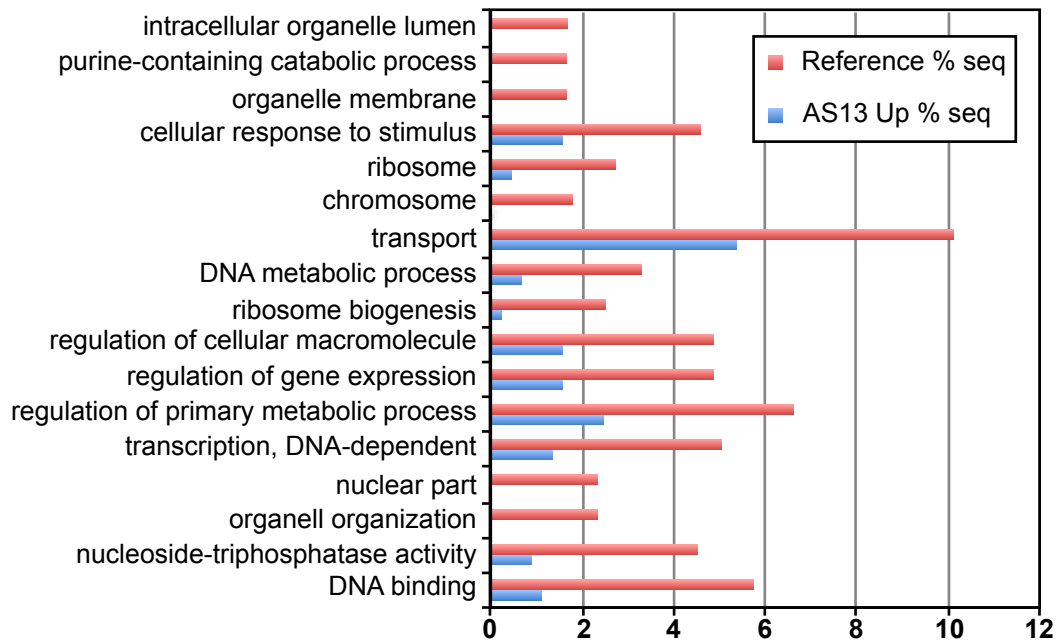

Supplement: Additional file 6: Figure S4. — Over- (a.) and under- (b.) represented Gene Ontology (GO) terms of R. solani genes up-regulated when confronted with S4 Serratia proteamaculans. [file 12864_2015_1758_MOESM6_ESM.zip › Figure S6b.pdf]

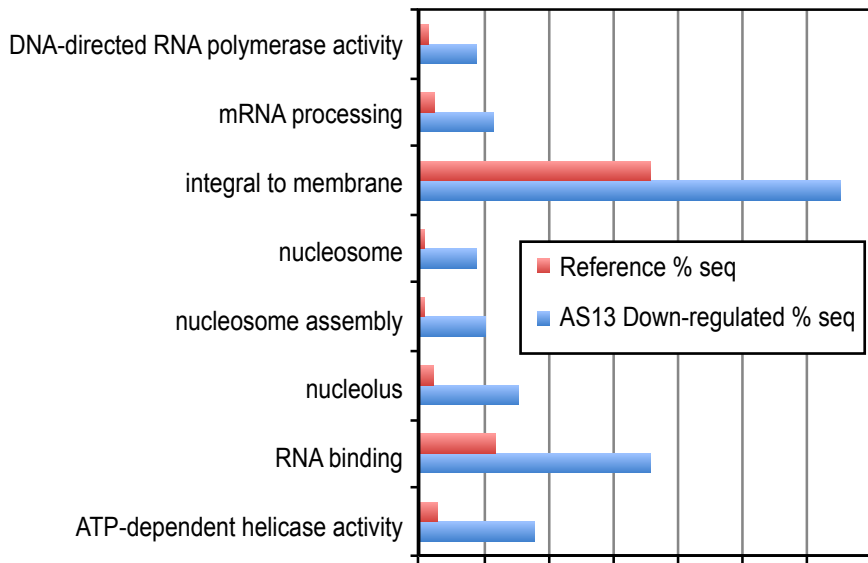

Supplement: Additional file 7: Figure S5. — Over- (a.) and under- (b.) represented Gene Ontology (GO) terms of R. solani genes down-regulated when confronted with S4 Serratia proteamaculans. [file 12864_2015_1758_MOESM7_ESM.pdf]

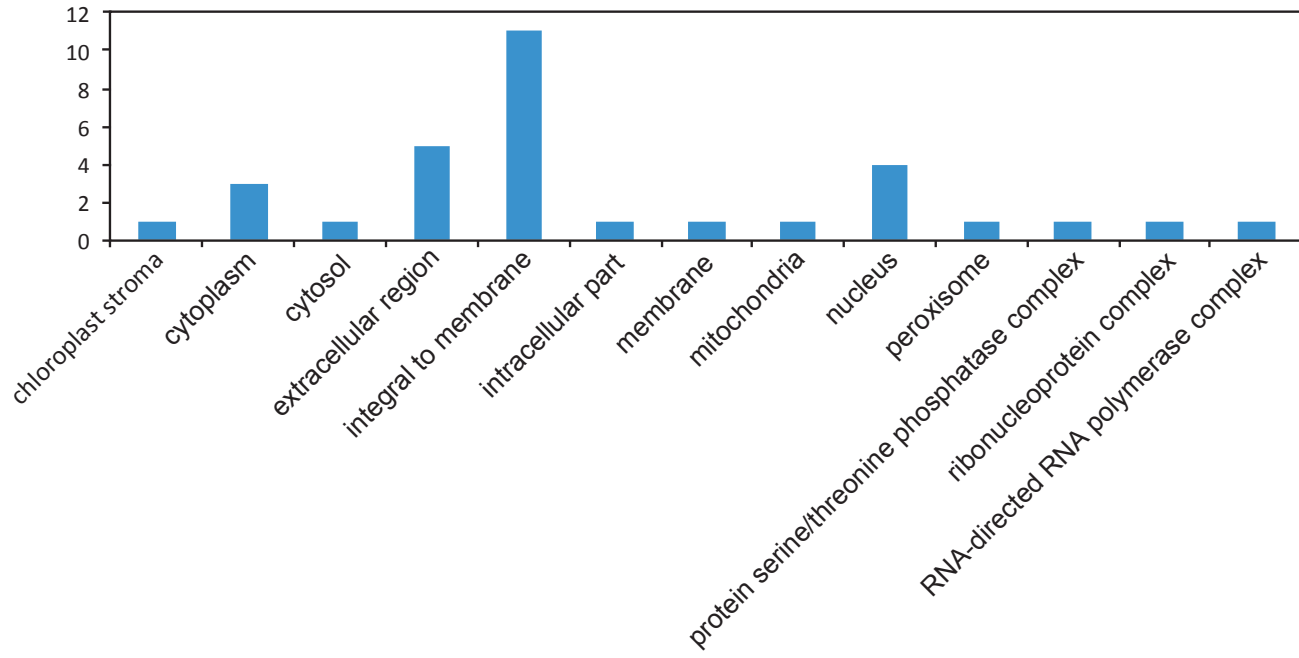

Supplement: Additional file 8: Figure S6. — Over- (a.) and under- (b.) represented Gene Ontology (GO) terms of R. solani genes up-regulated when confronted with AS13 Serratia plymuthica. [file 12864_2015_1758_MOESM8_ESM.zip › FIgure S8a.pdf]

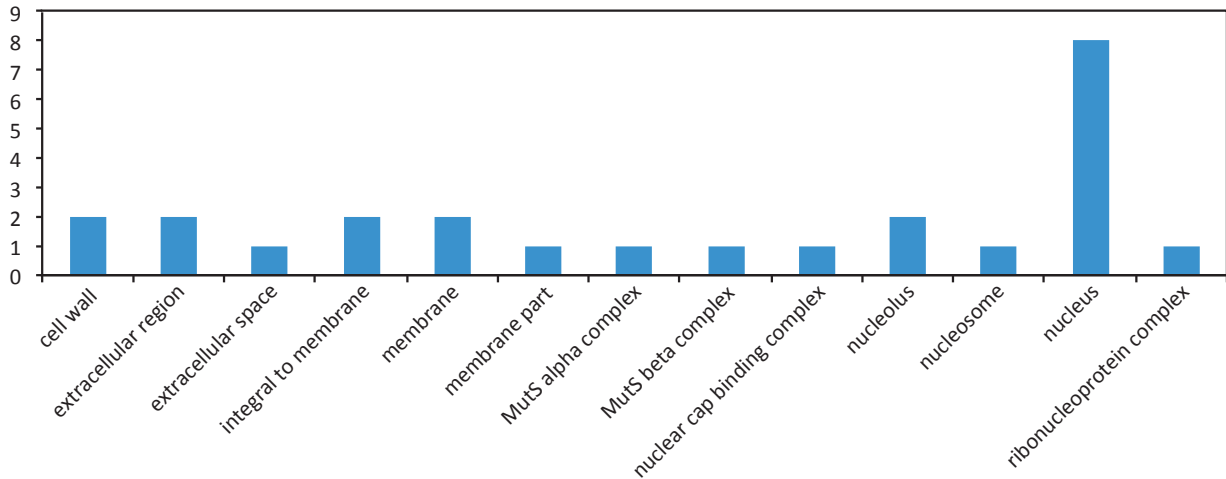

Supplement: Additional file 8: Figure S6. — Over- (a.) and under- (b.) represented Gene Ontology (GO) terms of R. solani genes up-regulated when confronted with AS13 Serratia plymuthica. [file 12864_2015_1758_MOESM8_ESM.zip › Figure S8b.pdf]

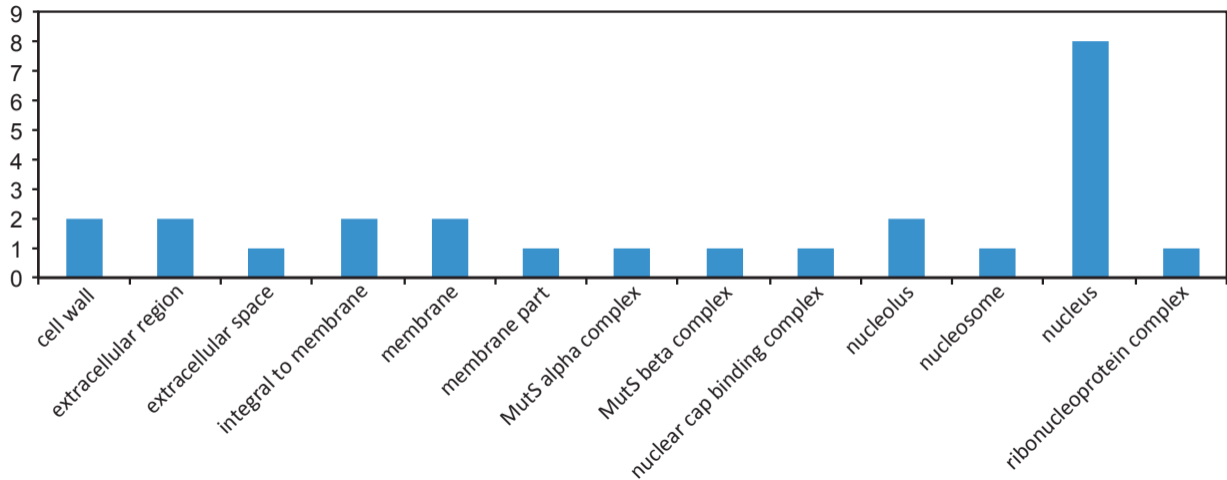

Supplement: Additional file 9: Figure S7. — Over-represented Gene Ontology (GO) terms of R. solani genes down-regulated when confronted with AS13 Serratia plymuthica. [file 12864_2015_1758_MOESM9_ESM.zip › Figure S9a.pdf]

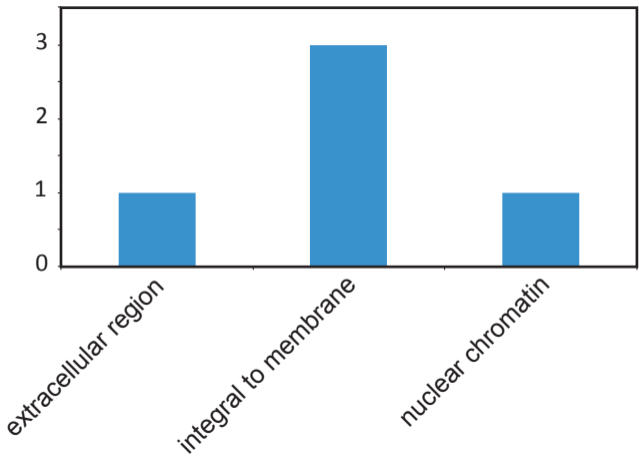

Supplement: Additional file 9: Figure S7. — Over-represented Gene Ontology (GO) terms of R. solani genes down-regulated when confronted with AS13 Serratia plymuthica. [file 12864_2015_1758_MOESM9_ESM.zip › Figure S9b.pdf]

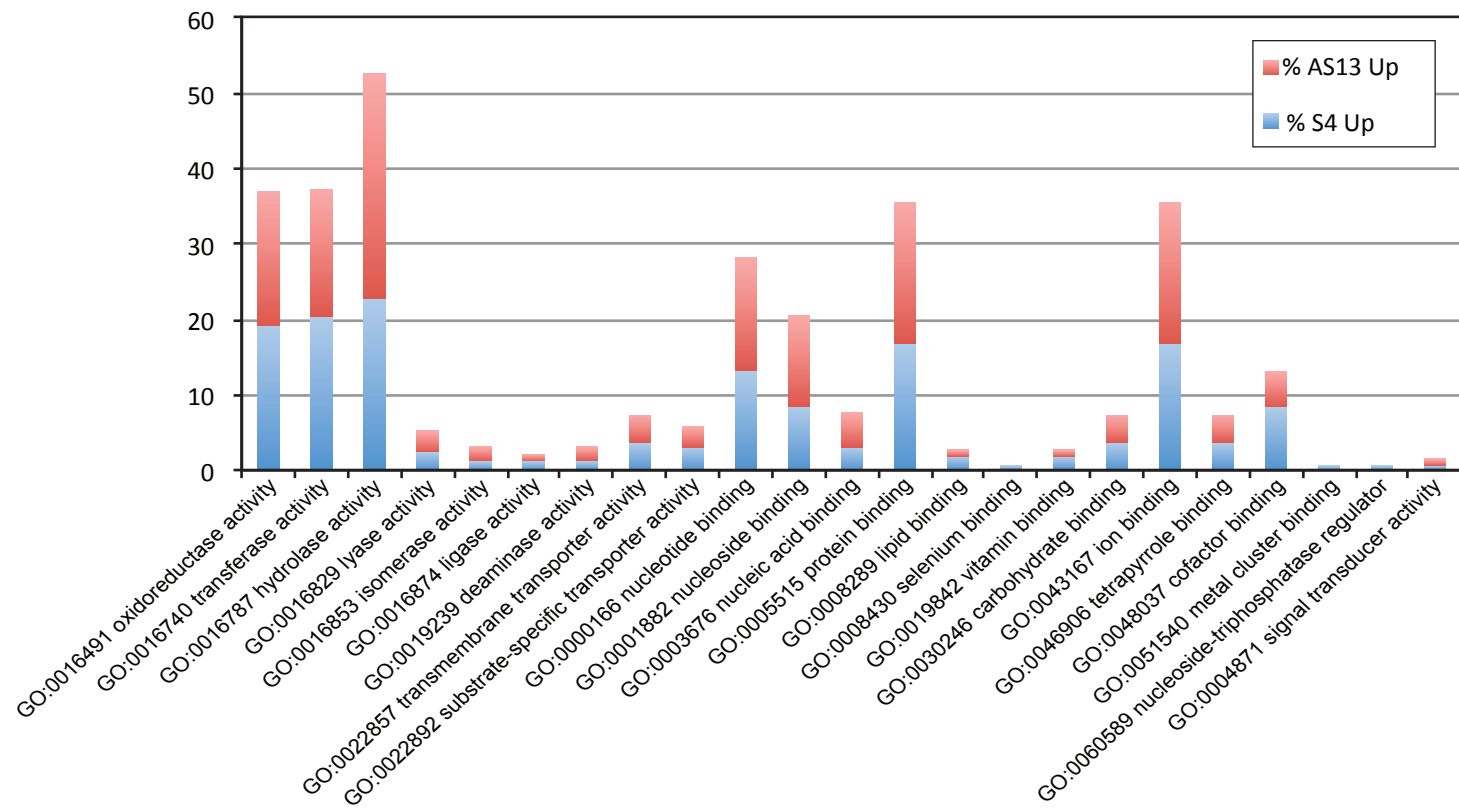

Supplement: Additional file 10: Figure S8. — Over-represented Cellular Location terms from Gene Ontology (GO) of R. solani genes up- (a.) and down- (b.) regulated when confronted with S4 Serratia proteamaculans. [file 12864_2015_1758_MOESM10_ESM.zip › Figure S10a.pdf]

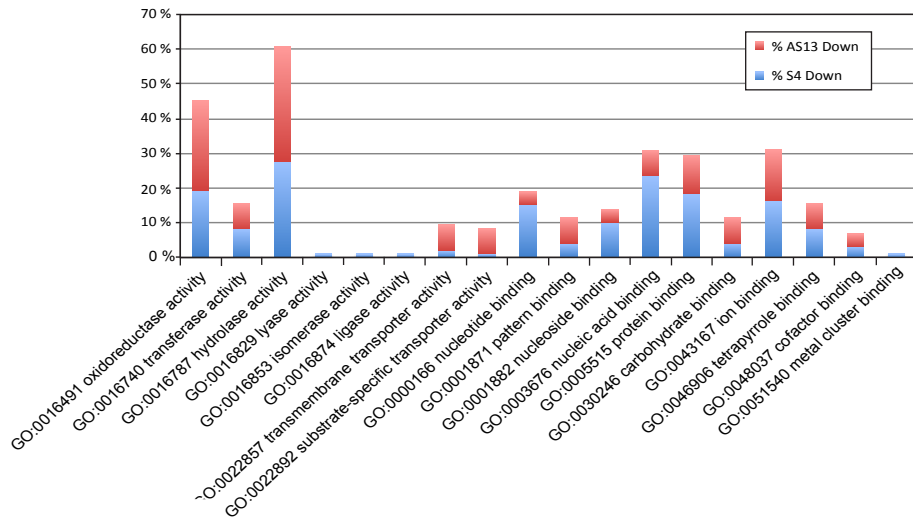

Supplement: Additional file 10: Figure S8. — Over-represented Cellular Location terms from Gene Ontology (GO) of R. solani genes up- (a.) and down- (b.) regulated when confronted with S4 Serratia proteamaculans. [file 12864_2015_1758_MOESM10_ESM.zip › Figure S10b.pdf]

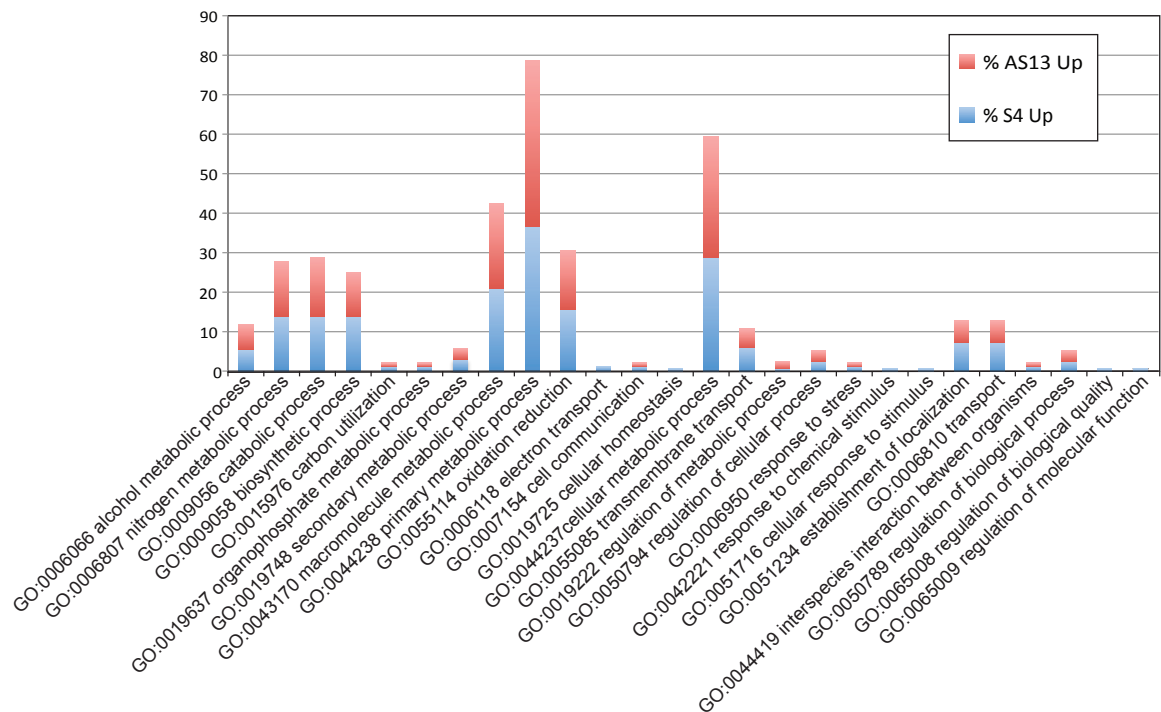

Supplement: Additional file 11: Figure S9. — Over-represented Cellular Location terms from Gene Ontology (GO) of R. solani genes up- (a.) and down- (b.) regulated when confronted with AS13 Serratia plymuthica. [file 12864_2015_1758_MOESM11_ESM.zip › Figure S11a.pdf]

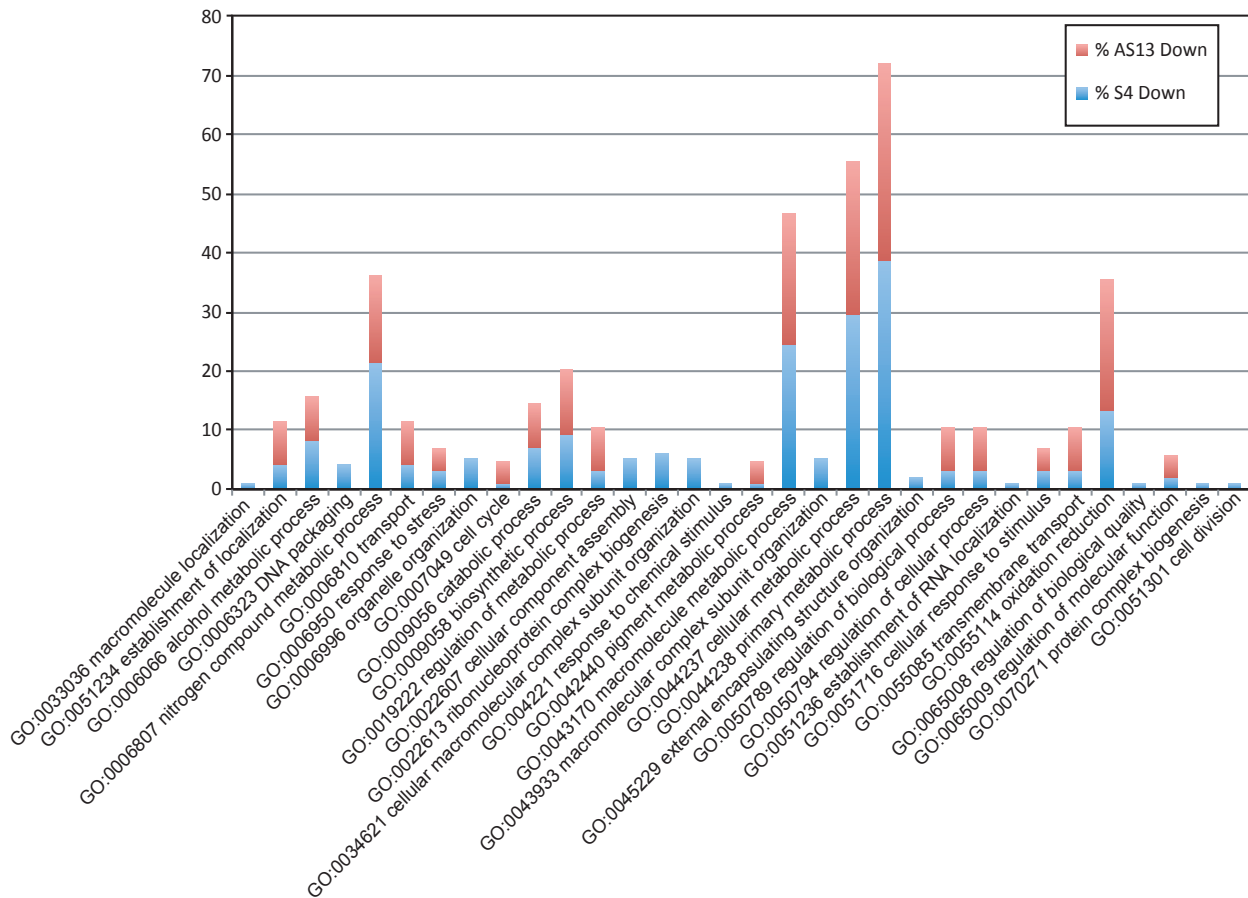

Supplement: Additional file 11: Figure S9. — Over-represented Cellular Location terms from Gene Ontology (GO) of R. solani genes up- (a.) and down- (b.) regulated when confronted with AS13 Serratia plymuthica. [file 12864_2015_1758_MOESM11_ESM.zip › Figure S11b.pdf]

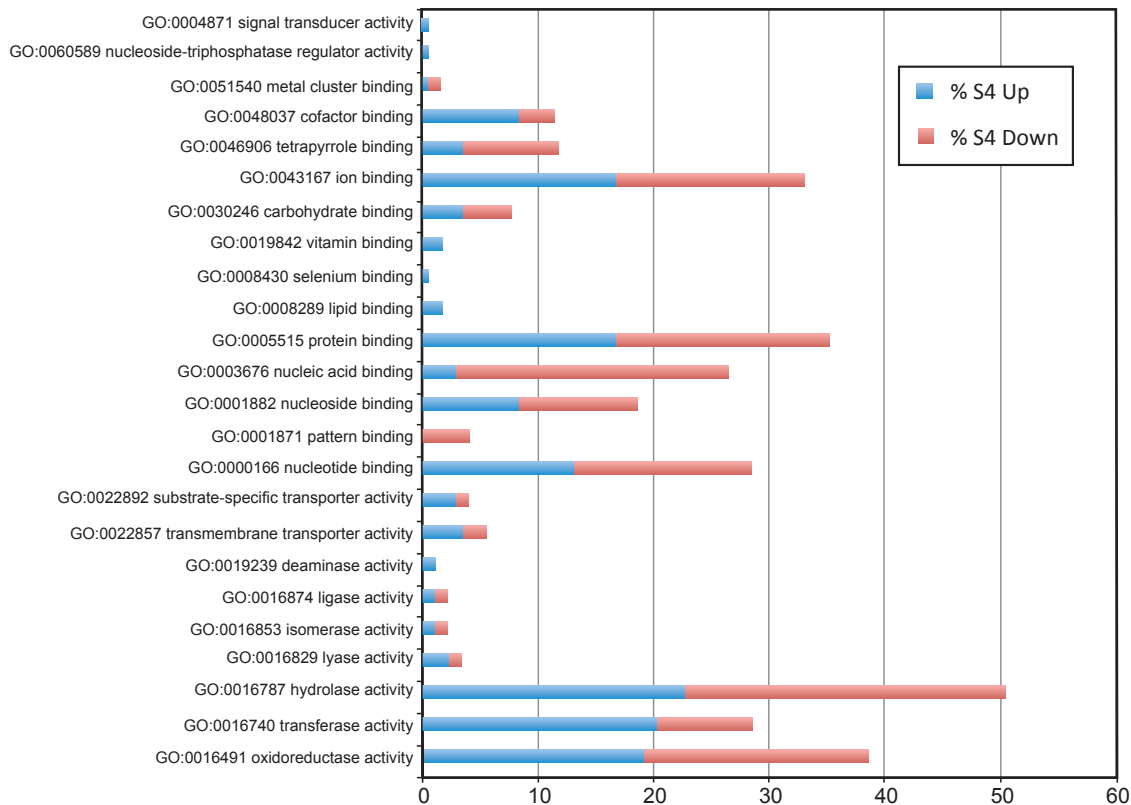

Supplement: Additional file 12: Figure S10. — Comparison of Molecular Function annotations of R. solani genes between S4 Serratia proteamaculans and AS13 Serratia plymuthica treatments. a) Bar Chart of Gene Ontology (GO) annotations between S4-AS13 upregulated genes, b) Bar Chart of GO annotations between S4-AS13 downregulated genes (Values taken from WEGO). [file 12864_2015_1758_MOESM12_ESM.pdf]

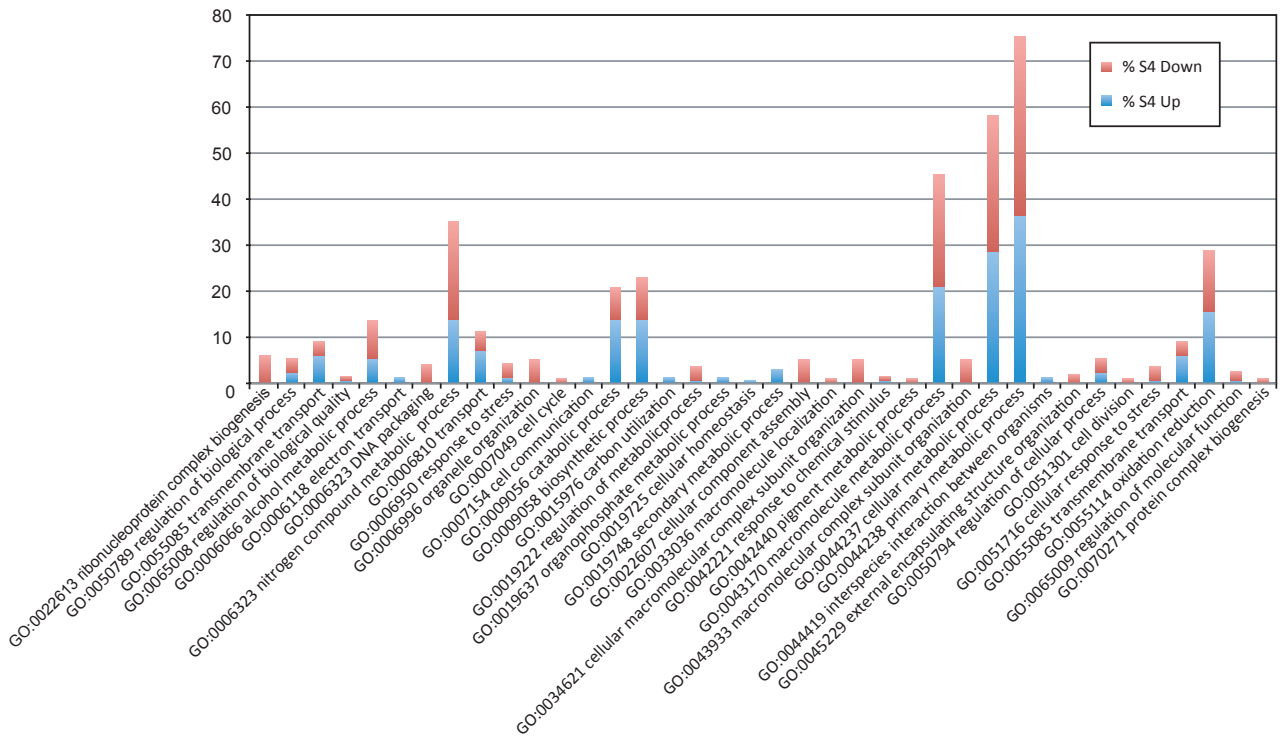

Supplement: Additional file 13: Figure S11. — Comparison of Biological Process annotations of R. solani genes between S4 Serratia proteamaculans and AS13 Serratia plymuthica treatments. a) Bar Chart of Gene Ontology (GO) annotations between S4-AS13 upregulated genes, b) Bar Chart of GO annotations between S4-AS13 downregulated genes (Values taken from WEGO). [file 12864_2015_1758_MOESM13_ESM.pdf]

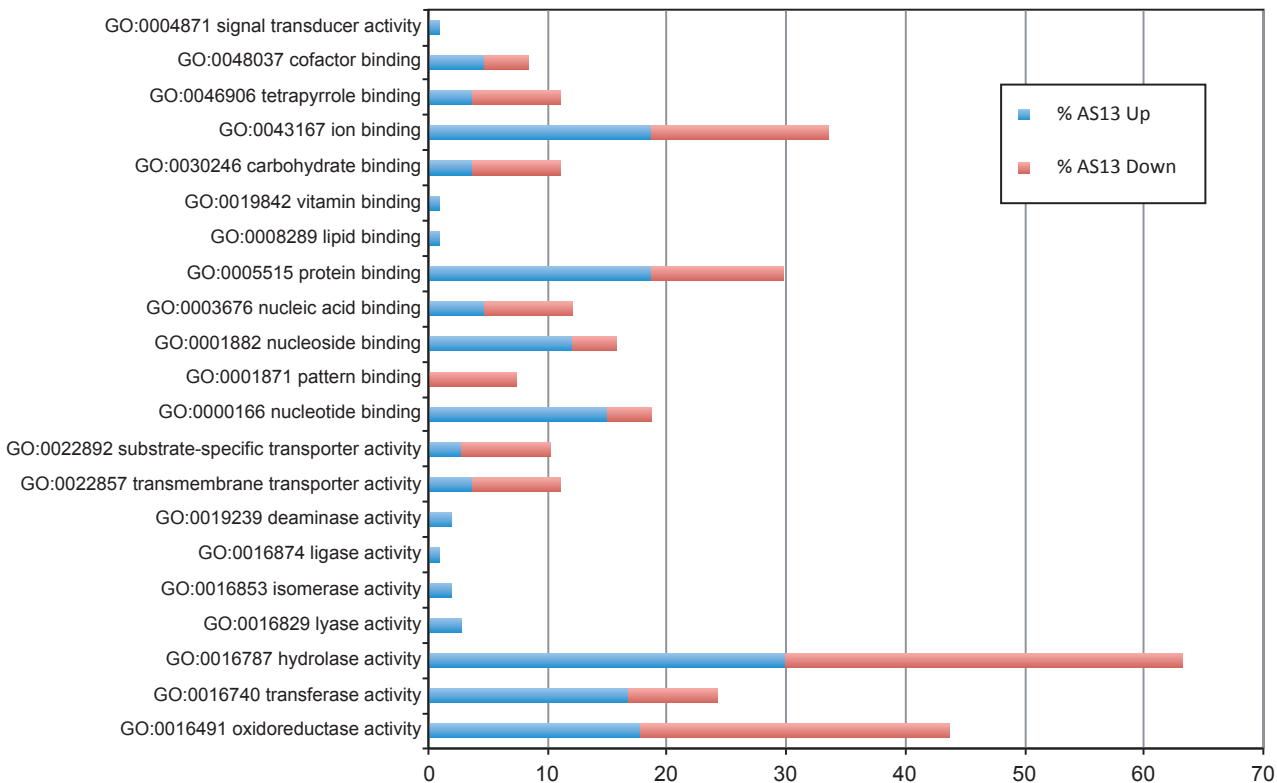

Supplement: Additional file 14: Figure S12. — Comparison of Molecular Function annotations of R. solani genes between the induced (log2FPKM > 0) and repressed (log2FPKM < 0) genes when challenged with S4 Serratia proteamaculans. (FPMK = fragments per kilobase of exon per million fragments mapped) [file 12864_2015_1758_MOESM14_ESM.pdf]

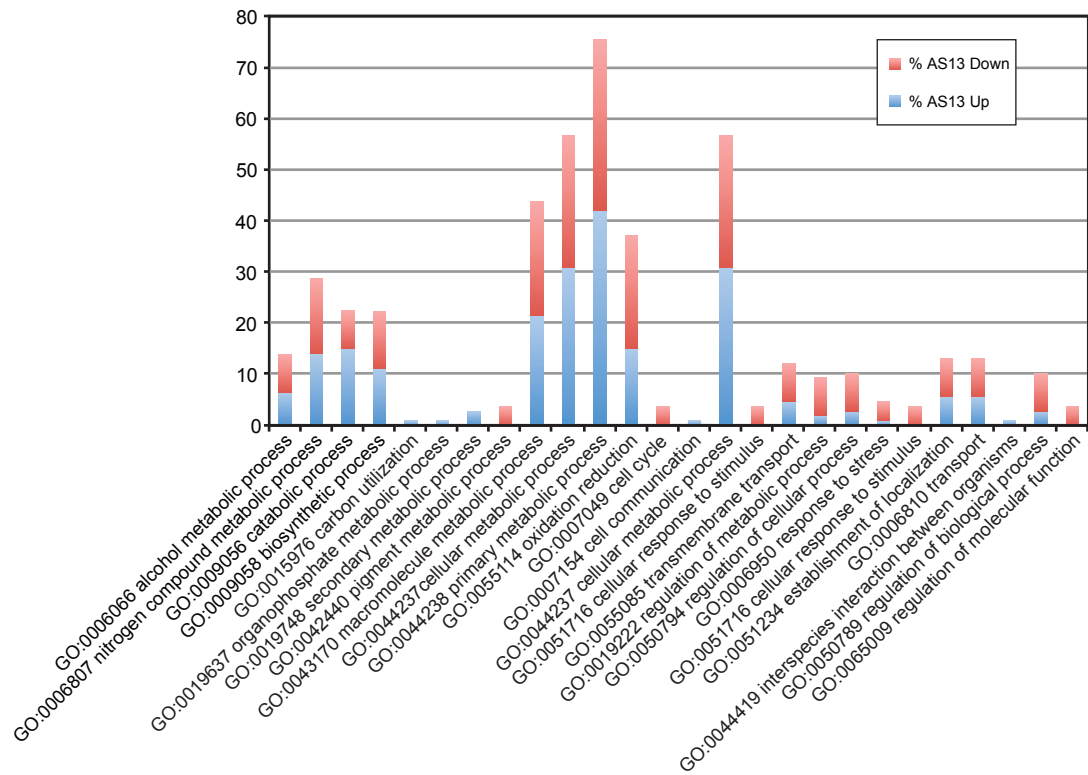

Supplement: Additional file 15: Figure S13. — Comparison of Biological Process annotations of R. solani genes between the induced (log2FPKM > 0) and repressed (log2FPKM < 0) genes when challenged with S4 Serratia proteamaculans. (FPMK = fragments per kilobase of exon per million fragments mapped) [file 12864_2015_1758_MOESM15_ESM.pdf]
